# Integrating Overall Water Splitting with Advanced Oxidation for Wastewater Treatment Using a Bifunctional Medium-Entropy Amorphous Alloy

**DOI:** 10.1007/s40820-026-02172-1

**Published:** 2026-04-16

**Authors:** Yifan Cui, Yonghui Wang, Bo Li, Jiaqi Huang, Le Bo, Hengqi Liu, Mahlanyane Kenneth Mathe, Murodjon Samadiy, Shengfeng Guo, Hongxian Shen, Jianfei Sun, Sida Jiang

**Affiliations:** 1 School of Materials Science and Engineering, Harbin Institute of Technology, Harbin, 150001 People’s Republic of China; 2 Laboratory for Space Environment and Physical Sciences, Harbin Institute of Technology, Harbin, 150001 People’s Republic of China; 3 Frontier Science Center for Interaction Between Space Environment and Matter, and National Key Laboratory of Space Environment and Matter Behaviors, Harbin, People’s Republic of China; 4 National Key Laboratory for Precision Hot Forming, Harbin Institute of Technology, Harbin, 150001 People’s Republic of China; 5 School of Physics, Harbin Institute of Technology, Harbin, 150001 People’s Republic of China; 6 Department of Chemistry, University of South Africa, Johannesburg, 1709 South Africa; 7 Department of Chemical Engineering and Biotechnology, Karshi State Technical University, 180100 Karshi, Uzbekistan; 8 School of Materials and Energy, Chongqing Key Laboratory for Advanced Materials and Technologies of Clean Energies, Southwest University, Chongqing, 400715 People’s Republic of China

**Keywords:** Medium-entropy amorphous alloy, Crystalline-amorphous heterostructure, Water splitting, Water remediation, Water–energy nexus strategy

## Abstract

**Highlights:**

- (FeCoNi)_80_B_20_ fibers were prepared via a melt-extraction method with a high cooling rate to construct a crystalline–amorphous heterostructure. A substantial quantity of fibers (~ 100 g) was produced in a single batch, resulting in a competitive cost of approximately 0.51 USD per gram with accurate composition and high quality.
- In simulated reclaimed water, complete decolorization of the solution was achieved within 60 min, whereas the water splitting process maintained stable operation for approximately 115 h. The total organic carbon removal rate reached 63.24%.

**Abstract:**

Hydrogen energy is regarded as a clean and reliable approach for storing intermittent energy sources. However, stringent water quality requirements remain critical challenges. The development of a bifunctional catalyst capable of simultaneously driving overall water splitting and degrading pollutants in wastewater can substantially enhance energy utilization efficiency and enable resource recycling. Nevertheless, the mismatch in the optimal pH conditions and the difficulty in balancing degradation efficiency and electrolysis performance remain notable obstacles. In this study, (FeCoNi)_80_B_20_ medium-entropy amorphous alloy (MEAA) fibers were prepared using a low-cost melt-extraction method. Owing to the crystalline–amorphous heterostructure, the fibers achieved complete decolorization within 90 s, while remaining effective across a wide pH range. In addition, the (FeCoNi)_80_B_20_ delivered overpotentials of 275 and 220 mV for the oxygen evolution reaction and hydrogen evolution reaction, respectively. By synchronizing both catalytic reactions, the (FeCoNi)_80_B_20_ enabled direct water splitting in reclaimed water, achieving complete decolorization while preserving electrocatalytic stability in an anion-exchange-membrane electrolyzer for 100 h under highly alkaline conditions (pH = 13.6). Moderate OH* adsorption endowed (FeCoNi)_80_B_20_ with excellent ability. This bifunctional catalyst addresses the coupled challenges of energy storage and water scarcity and offers a promising foundation for industrial implementation.
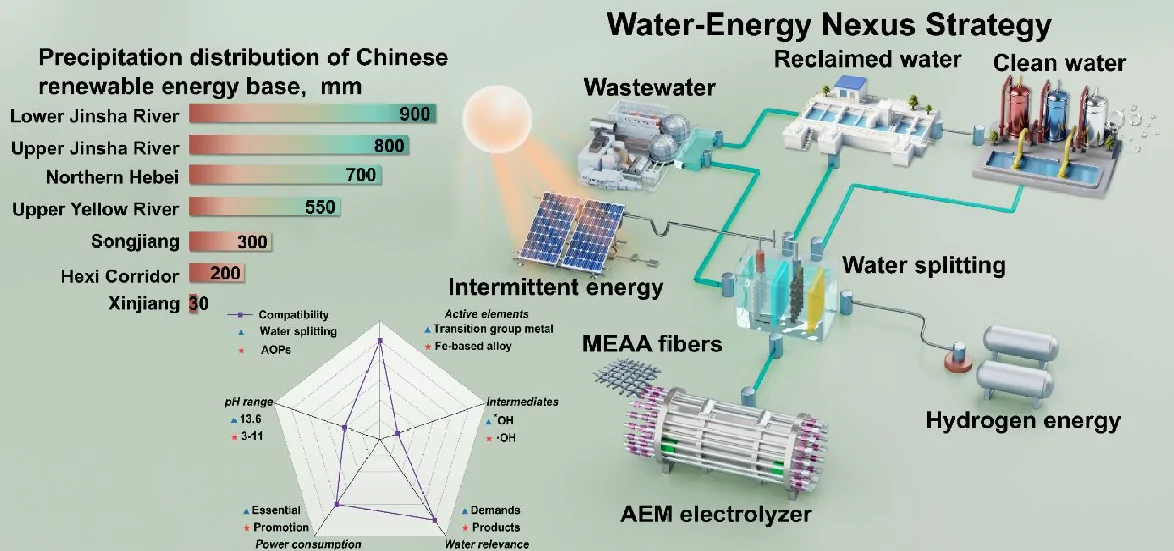

**Supplementary Information:**

The online version contains supplementary material available at 10.1007/s40820-026-02172-1.

## Introduction

The scarcity of energy resources presents a significant obstacle to achieving sustainable development in modern society [1–3]. The electrolysis of water for hydrogen production serves as a highly promising energy storage method and has been widely employed to store intermittent energy sources such as wind and solar power [4–7]. Water electrolysis involves the oxygen evolution reaction (OER) at the anode and the hydrogen evolution reaction (HER) at the cathode [8–10]. Compared to the HER, the OER exerts a more significant influence on the overall efficiency of water splitting, due to its higher energy barrier and the intrinsic complexity of the four-electron transfer process [11, 12]. During the OER, oxygen intermediates (OI) (e.g., OH*, O*, and OOH*) are formed, among which the adsorption strength of OH* is generally considered a key factor governing catalytic activity [13, 14]. A moderate adsorption strength for OI is crucial for enhancing the overall water splitting activity of a catalyst. Significant research in recent years has focused on developing electrocatalysts for water splitting, with particular emphasis on reducing the overpotential (*ŋ*) [15–17], lowering costs [18–20], and enhancing stability [21, 22]. Various oxides [23–26], hydroxides [27, 28], and transition metal alloys [29–34] have demonstrated excellent performance in water splitting. In practical industrial applications, the operating performance of electrolyzers and the purity of the produced gases are strongly influenced by the quality of the feed water [35, 36]. Meanwhile, regions hosting large-scale wind and solar energy harvesting systems frequently suffer from water scarcity [37, 38]. Consequently, improving the stability of electrocatalysts under fluctuating current densities supplied by intermittent energy sources, while mitigating the adverse effects of water quality on catalytic performance, is pivotal for the practical deployment of hydrogen production via water splitting as an energy storage route.

Wastewater treatment represents an effective strategy for improving water utilization efficiency [39–41]. For instance, China’s chemical oxygen demand (COD) discharge in wastewater reached 2.9544 × 10^7^ metric tons in 2023. Wastewater treatment technologies can be broadly classified into microbial [42], physical [43], and chemical processes [44]. Notably, advanced oxidation processes (AOPs) have attracted considerable attention because of their broad industrial applicability and relatively low cost [45, 46]. The mechanism involves a catalytic chain reaction between the catalyst and oxidant. The reaction generates highly oxidative hydroxyl radicals (·OH), which enable the degradation of diverse toxic and recalcitrant organic compounds for effective wastewater treatment. Typical AOPs include electrochemical catalytic oxidation, photocatalytic oxidation, wet oxidation, and Fenton/Fenton-like processes [47, 48]. The Fenton and Fenton-like methods are particularly attractive due to their high organic degradation efficiency, absence of secondary pollution, and wide applicability [49, 50]. To reduce operational costs and extend reuse cycles, iron-based alloys have been extensively investigated as substitutes for conventional industrial iron salts [51]. However, issues such as the restriction to acidic environments, limited reusability, and relatively slow degradation kinetics remain unresolved.

If water splitting and pollutant degradation can be integrated and conducted directly in wastewater, energy storage costs can be reduced while simultaneously enabling water resource recycling. Recent studies have used of seawater [52–54] or municipal reclaimed water [55] after certain treatment to achieve water splitting. However, incomplete water degradation prevents the further recycling of water resources. In studies of OER catalysis, Liu et al. employed methanol as a probe molecule to investigate the influence of OH* adsorption strength on catalytic activity [56], explicitly defining OH* electrophilicity as being bounded by the ·OH as the upper limit and the hydroxide anion (OH⁻) as the lower limit. The strong adsorption of OH* would improve the degradation efficiency of AOPs and increase the kinetic barrier of OER [57–59], suggesting that modulation of OH* adsorption strength is a feasible strategy for the development of bifunctional catalysts capable of operating in a synchronized process of AOPs and water splitting. A strong adsorption of OH* promotes its conversion to ·OH radicals, thereby improving the degradation rate. However, excessively strong adsorption of OH* can compromise the catalytic activity for OER. Therefore, appropriately balancing the performance of both reactions is key to developing bifunction catalysts. However, substantial challenges remain in the design of dual-function catalysts and pH regulation to enable the efficient catalysis of both reactions, thereby achieving effective conversion and recycling of energy and water resources.

Therefore, a dual-function catalyst capable of simultaneously enabling advanced oxidation and water splitting was proposed in this study. By exploiting the synergistic effects of multi-principal elements and regulating the adsorption strength for both catalytic reactions, (FeCoNi)_80_B_20_ medium-entropy amorphous alloy (MEAA) fibers were fabricated via a melt-extraction method with rapid cooling at a low material cost (~ 0.51 USD per gram). Owing to the crystalline–amorphous (*c-a*) heterostructure, the fibers exhibited exceptional degradation performance, achieving complete dye removal within 90 s and maintaining catalytic activity over more than 30 reuse cycles, while remaining effective across a broad pH range. Meanwhile, the (FeCoNi)_80_B_20_ MEAA demonstrated excellent water splitting activity, delivering *ŋ*_*10*_ values of 275 and 220 mV for OER and HER, respectively. When employed as both anode and cathode, the electrodes maintained stable operation for 144 h under practical fluctuating current densities. In the electrolytes containing Rhodamine B (RhB), water splitting accelerated the oxidation of Fe^0^ to active Fe^3+^ species and enhanced electron transport, thereby promoting faster dye degradation while preserving sufficient stability during electrolysis. Furthermore, a flow-type anion-exchange-membrane (AEM) electrolyzer assembled with (FeCoNi)_80_B_20_ MEAA as both electrodes achieved a current density of 200 mA cm^−2^ at approximately 1.96 V for over 100 h in reclaimed water, albeit with a moderate voltage increase. Structural characterization combined with density functional theory (DFT) calculations revealed that the *c-a* heterostructure could provide abundant active sites, whereas Fe, Co, and Ni collectively regulated OH* adsorption. Moderate OH* adsorption facilitated ·OH generation at the anode during water electrolysis, thereby enhancing degradation efficiency. The development of this bifunctional catalyst for AOPs and water splitting reduces the cost of water electrolysis for energy storage and enables resource recycling, highlighting its promising potential for industrial applications.

## Experimental Section

### Preparation of MEAA Fibers

As-cast melt-extracted was used for manufacturing a range of fibers. According to the nominal composition, a total mass of ~ 120 g of starting materials was mixed using Fe (99.99%), Co (99.99%), Ni (99.995%), and B (99.99%). These elemental powders were provided by the ZhongNuo Advanced Material Technology Co. Ltd., China. Analytical-grade chemical reagents were all utilized immediately, without any additional purification. The beginning materials were smelted 4–5 times using vacuum arc smelting to guarantee that the components are evenly combined while being shielded by high-purity argon (99.999%). Then, the metal rod (about 2–3 cm) was melted in a high-purity BN crucible, and the resulting solution was dipped in oxygen and discarded as soon as the copper wheel began to rotate and met the molten surface. The alloy rod was melted by applying a current of approximately 22.5 A and then fed toward the copper wheel at a constant speed of 30 μm s^−1^. The copper wheel rotated at a surface velocity of 1700 r min^−1^. Upon contact with the wheel, the molten alloy underwent rapid solidification due to the large temperature gradient and was subsequently spun off to form continuous fibers. Ultimately, HEA fibers measuring around 3–4 cm in length, 30–35 µm in diameter, and ~ 115 g in total mass were produced. Among all the elements, aluminum corrodes the easiest. To increase the specific surface area, porous morphological characteristics were realized via a typical dealloying corrosion strategy in 1.0 M HCl. After dealloying, the as-obtained fibers were successively cleaned by absolute ethyl alcohol (99.5%) and distilled water (18.2 MΩ cm) for 30 min. The mixing entropy of the alloys was calculated by using:

1 $$\Delta {S}_{mix}=-R\sum {x}_{i}\mathrm{ln}{x}_{i}$$

where *R* is the gas constant and *x*_*i*_ is the atomic (or mole) fraction of component.

The mixing entropy of (FeCoNi)_80_B_20_ is approximately 1.38R that meet the definition of medium-entropy alloys [60, 61].

### Physical Characterization

The morphology of fibers was characterized by field-emission scanning electron microscopy (FE-SEM) and transmission electron microscopy (TEM). Scanning electron microscopy (SEM) observations were made using the Carl Zeiss Merlin Compact and TEM analysis was conducted using an FEI Talos F200s, operating at 200 kV. The electron back scatter Raman spectra were collected using a LabRAM HR Evolution. X-ray photoelectron spectroscopy (XPS) was carried out using a ThermoFisher Scientific Esca Xi + , equipped with Al Kα radiations (1486.6 eV). The inductively coupled plasma-optical emission spectrometry (ICP-OES) was measured using Thermo iCAP 740.

### Catalytic Experiments of Degradation

Both as-prepared fibers were employed as catalysts for PS activation. RhB, SM2, and benzothiazole were used as target pollutants, and residual concentrations were measured via high-performance liquid chromatography (HPLC Scion LC6000). The detection wavelengths were 550 nm for RhB, 270 nm for SM2, 270 nm for phenol, and 245 nm for benzothiazole, respectively, with mobile phase as follows: methanol and 0.1% formic acid solution (70: 30, V: V), acetonitrile and 0.1% formic acid solution (30: 70, V: V), and methanol and 0.1% formic acid solution (45: 55, V: V), respectively. Unless stated otherwise, the catalyst amount was 0.5 g L^−1^, pollutant and PS concentration were 25 mg L^−1^ and 10 mm, respectively. Moreover, experiments were conducted at ambient temperature with a pH of 5.0. For each test, 100 mL of aqueous pollutant solution was prepared, and catalysts were dispersed within it. Upon the addition of PS, the reaction start time was recorded. Samples of 4 mL were collected at predetermined intervals, passed through a 0.22 μm pore-sized filter membrane, and analyzed using HPLC and TOC measurements. The kinetic rate constant (k_obs_) was calculated by using:

2 $$\mathrm{ln}({C}_{0}/{C}_{t})={k}_{obs}$$

where *C*_*0*_ is the original concentration of the pollutant and *C*_*t*_ is the concentration of the pollutant at time t.

To evaluate catalyst stability, samples underwent ultrasonic cleaning in ultrapure water and ethanol between cycles and reused. Radical types were confirmed using an ESR/EPR (Brook EMX Plus).

### Electrochemical Measurements

Electrochemical measurements were conducted using an electrochemical working station (CHI 760E) in a three-electrode setup, where 1 M KOH solution was used as an electrolyte, an Hg/HgO electrode (in 1 M KOH) was used as a reference electrode and the graphite rod (99.9%, with a diameter of 8 mm) was used as a counter electrode. Oxygen evolution reaction (OER) overpotential (*ŋ*) was calculated using the following relationship:

3 $$ {\mathrm{E}}\left( {{\mathrm{RHE}}} \right) = {\mathrm{E}}\left( {{\mathrm{Hg}}/{\mathrm{HgO}}} \right) + 0.0{\mathrm{59pH}} + 0.0{98} $$

LSV and Tafel curves were corrected by 95% iR compensation for the ohmic loss except for AEM test. Electrochemical impedance spectroscopy (EIS) was carried out at 1.58 V in the frequency range of 1000 kHz to 10 mHz with an amplitude of 5 mV. EIS analysis was carried out by fitting the Nyquist plot using an equivalent circuit, which was realized by the Z-view software. Chronopotentiometry measurements were performed to evaluate the long-term stability. The electrochemical surface area (ECSA) was determined by measuring the capacitive current associated with double-layer charging from five different scan-rates (10, 30, 50, 70, 90, and 110 mV s^−1^). In these regions, the integrated charge should be due to the charging of the electrode–electrolyte double layer. The double-layer charging current (i), normalized on the geometric area of the electrode, is directly proportional to the scan rate (υ), i.e., i = υC_dl_. By drawing the curves of anodic and cathodic currents with respect to the scan rate, the average C_dl_ of linear fitting slope is derived. The measured capacitance currents are plotted as a function of the scan rate, as shown in Fig. S15. According to the reported typical value, the specific capacitance (C_s_) of electrocatalyst in 1 M KOH is selected as C_s_ = 0.040 mF cm^2^. Therefore, the ECSA of the catalyst layer can be calculated using Eq. (4):

4 $$ {\mathrm{ECSA}} = {\mathrm{C}}_{{{\mathrm{dl}}}} /{\mathrm{C}}_{{\mathrm{s}}} $$

About 10 fibers were arranged in parallel function and employed as catalyst materials for the anode and cathode in AEM. The active area corresponds to the collective geometric surface area of the fibers (assumed as cylinder). The electric conduction and electrolyte transport are performed by S-type Ti current collectors with a serpentine flow field. The AEM electrolyzer was put together with the cathode or anode compartment first, followed by the end plate, sealing gasket, Ti current collector, fibers, and sealing gasket. An anion-exchange membrane (Fumasep FAA-3-PK-130) was employed to divide the electrolyzer’s cathode and anode compartments. A peristaltic pump was used to regulate the flow of the simulated wastewater (1.0 M KOH with 25 mg L^−1^ RhB) electrolyte to both sides of the electrolyzer at a rate of 2.5 mL min^−1^ throughout the testing.

### Theoretical Calculations

All the calculations are performed in the framework of the density functional theory with the projector augmented plane wave method, as implemented in the Vienna ab initio simulation package (VASP). The generalized gradient approximation (GGA) proposed by Perdew, Burke, and Ernzerhof (PBE) is selected for the exchange–correlation potential. The long-range van der Waals interaction is described by the DFT-D3 approach. The cutoff energy for plane wave is set to 480 eV. The energy criterion is set to 10 − 4 eV in iterative solution of the Kohn–Sham equation. All the structures are relaxed until the residual forces on the atoms have declined to less than 0.05 eV Å^−1^. Data analysis and visualization are carried out with the help of VASPKIT6 code and VESTA7. A 2 × 2 × 1 k-point mash in Brillouin zone was applied in this case. The system was equilibrated and analyzed in the NVT ensemble (constant particle number, volume, and temperature) at a temperature of 1000 K. A time step of 3 fs was employed to ensure accurate integration of the equations of motion. To avoid interlaminar interactions, a vacuum spacing of 20 Å is applied perpendicular to the slab.

Here, differences in Gibbs free energy (ΔG) for intermediates defined as:

5 $$ \Delta {\mathrm{G}} = \Delta {\mathrm{E}} + \Delta {\mathrm{EZPE}} - {\mathrm{T}}\Delta {\mathrm{S}} $$

where ΔG is the total energy difference between the slab and respective terminations computed by DFT-PBE. ΔEZPE and TΔS denote differences in zero-point energy and entropy between adsorbed states of reaction intermediates and gap phase, respectively. T is the room temperature (298.15 K).

## Results and Discussion

### Exploration of Combination of AOPs and Water Splitting

**Fig. 1:**
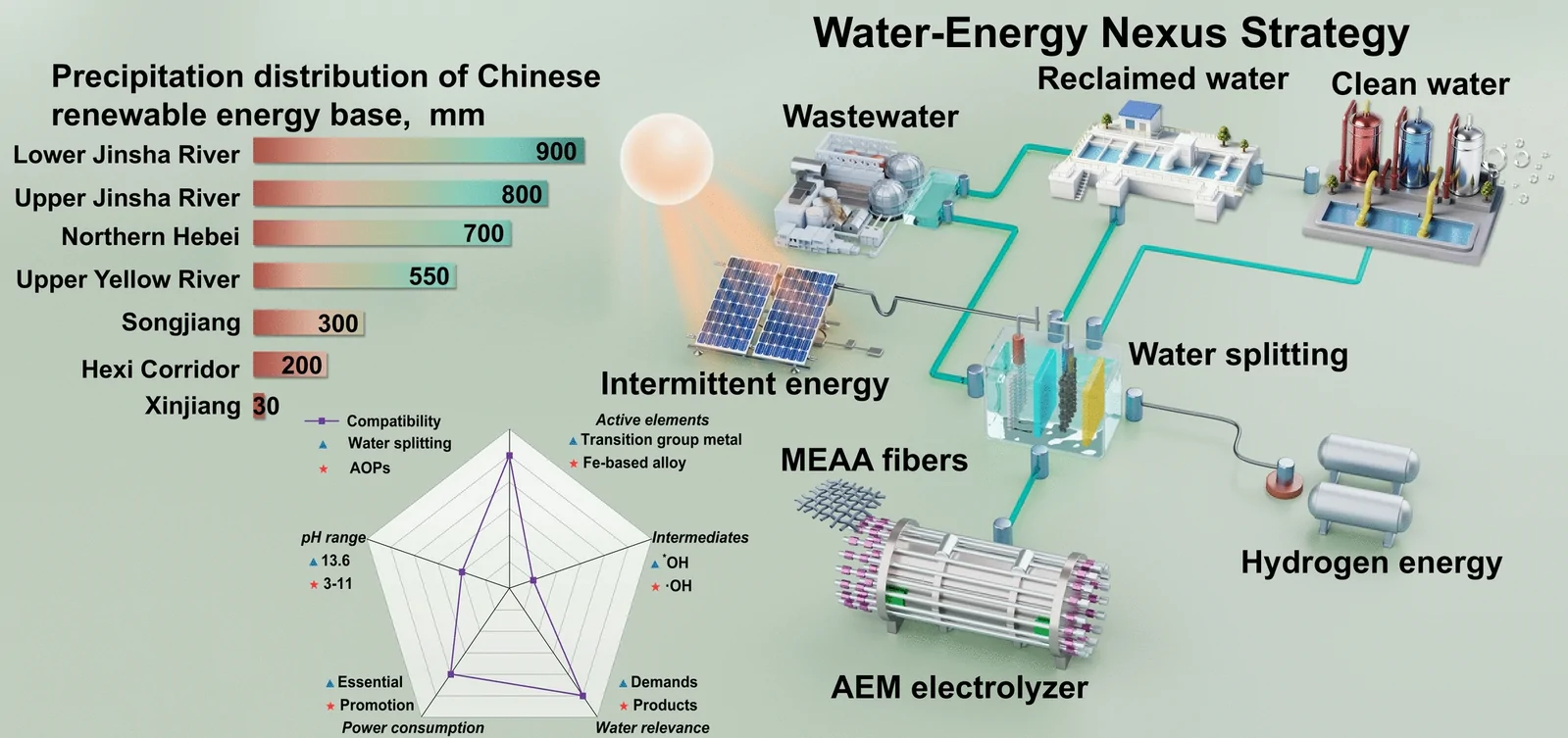
Conceptual illustration of the water–energy nexus strategy. Regions with large-scale solar and wind energy harvesting systems often experience severe water scarcity. The proposed system integrates energy storage and water purification through water splitting, coupled with advanced oxidation processes (AOPs). When using water electrolysis for hydrogen production to store intermittent energy sources, wastewater and reclaimed water can be directly introduced into the electrolyzer after simple physical filtration and adsorption. This integrated process enables stable oxygen evolution while simultaneously achieving effective water purification, thereby enhancing energy utilization efficiency and resource recycling. Inset: Radar chart illustrating the compatibility between water splitting and AOPs. Water splitting and AOPs exhibit compatibility in aspects active elements, intermediates, water relevance, power consumption, and pH range. This compatibility enables the integration of both reactions and suggests a design strategy for dual-function catalysts

In industrial applications, water electrolysis for hydrogen production represents an essential route for energy storage. However, water splitting is constrained by high energy consumption and stringent water quality requirements, while regions characterized by large-scale deployment of wind and solar energy harvesting systems often experience persistent water scarcity, thereby increasing costs and hindering practical implementation (Fig. 1). Hence, we proposed a “water-energy nexus” strategy. Performing overall water splitting directly in wastewater or reclaimed water while simultaneously achieving pollutant degradation can markedly enhance energy utilization efficiency and promote resource recycling. Accordingly, the combination of AOPs with water splitting is considered promising for several reasons (inset in Fig. 1). (1) Water relevance: The treated water can be directly reused for electrolytic hydrogen production. (2) Power consumption: The electrical energy applied during electrolysis can accelerate degradation kinetics. (3) Active elements: The active sites for both catalytic reactions can be primarily associated with transition metals. (4) Intermediates: Similar reaction intermediates can be involved and mutually transformed by regulating OH* adsorption. Although the optimal pH ranges for the two reactions are slightly different and require further optimization, this integrated route offers a practical strategy for stable storage of intermittent renewable energy in the form of hydrogen while enabling the direct utilization of reclaimed water. Consequently, the central challenge lies in the design of a bifunctional catalyst for AOPs and water splitting that delivers high catalytic performance under suitable pH conditions and a balance of activity for AOPs and water splitting. In this context, (FeCoNi)_80_B_20_ MEAA are regarded as promising bifunctional catalysts [62–64], as they contain Fe and Co active sites favorable for AOPs, along with Fe, Co, and Ni sites that are active for water splitting. The OH* binding strength follows the order Ni > Co > Fe [56]. The simultaneous incorporation of Fe, Co, and Ni enables the modulation toward a moderate OH* adsorption strength, thereby imparting bifunctional catalytic capability of AOPs and water splitting to (FeCoNi)_80_B_20_ in a synchronized process. In addition, the introduction of small-radius B enhances the glass-forming ability of MEAA, contributing to broadening the applicable pH window for catalytic operation.

### Electrocatalyst Synthesis and Characterization

**Fig. 2:**
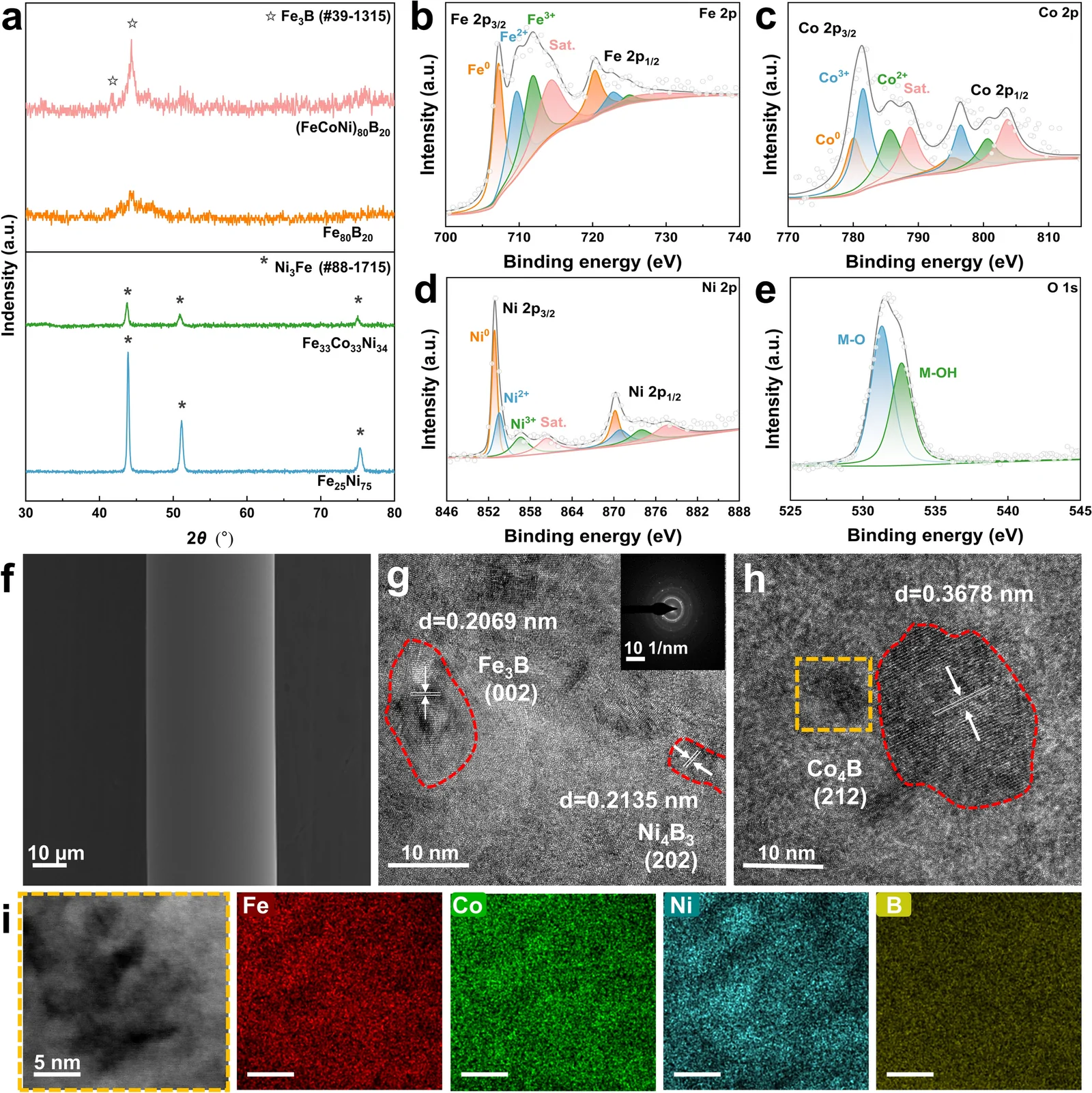
Structural characterization of (FeCoNi)_80_B_20_ ME-MGs. **a** X-ray diffraction patterns of Fe_80_B_20_, Fe_75_Ni_25_, Fe_33_Co_33_Ni_34_, and (FeCoNi)_80_B_20_. XPS spectra of **b** Fe 2*p*, **c** Co 2*p*, **d** Ni 2*p*, and **e** O 1*s*. **f** SEM image of the as-prepared fibers. **g** HRTEM image (inset: corresponding SAED pattern with polycrystalline rings), where a red dashed circle is used to highlight the nanocrystallites of Fe and Ni borides that are approximately 10–15 nm in size. **h** HRTEM image of Co boride nanocrystallites with a size of approximately 20 nm. **i** EDS analysis of the yellow square frame region in **h**

(FeCoNi)_80_B_20_ fibers along with a series of control samples were prepared via a melt-extraction method with a high cooling rate to construct a *c-a* heterostructure [65–68]. A substantial quantity of fibers (~ 100 g) was produced in a single batch (Fig. S1), resulting in a competitive cost of approximately 0.51 USD per gram with accurate composition and high quality [69–73], and indicating considerable potential for industrial application. Scanning electron microscopy (SEM) images revealed that the (FeCoNi)_80_B_20_ MEAA fibers possessed diameters of approximately 30 µm with smooth surfaces, while energy-dispersive X-ray spectrometry (EDS) mapping confirmed a uniform elemental distribution on the surface (Figs. 2f and S2). The X-ray diffraction (XRD) patterns (Fig. 2a) exhibited evident structural differences among the samples. Fe_80_B_20_ displayed a single broad diffraction peak characteristic of an amorphous structure. In contrast, the XRD pattern of (FeCoNi)_80_B_20_ demonstrated sharp nanocrystalline peaks corresponding to Fe_3_B (ICDD-PDF-2, PDF card no. 39–1315) and an amorphous feature still exists on the XRD patterns, indicating the construction of a partially crystallized state (or amorphous/crystalline structure) [74]. The Fe_25_Ni_75_ and Fe_33_Co_33_Ni_34_ exhibited crystalline structures with a characteristic Ni_3_Fe phase (ICDD-PDF-2, card no. 88–1715). The surface chemical states of the as-prepared (FeCoNi)_80_B_20_ were examined using X-ray photoelectron spectroscopy (XPS). The Fe 2*p* spectrum was deconvoluted into Fe^0^, Fe^2+^, and Fe^3+^ components, with Fe 2*p*_*3/2*_ binding energies of 707.2, 709.7, and 711.9 eV, respectively (Fig. 2b). Compared with Fe^2+^ and Fe^3+^, the relatively higher proportion of Fe^0^ could facilitate electron transfer and enhance the catalytic performance during degradation. The Co 2*p* spectrum was resolved into Co^0^, Co^2+^, and Co^3+^ species, with corresponding 2*p*_*3/2*_ binding energies of 780.1, 781.4, and 785.7 eV (Fig. 2c). For the active Ni sites associated with water splitting, Ni^0^ was predominant (Fig. 2d), suggesting the transformation to catalytically active Ni^3+^ during operation. The O 1*s* spectrum indicated the presence of M–O and M-OH bonds (Fig. 2e), while the B 1*s* spectrum confirmed the existence of B-B and B-O bonds (Fig. S3).

Cross-sectional transmission electron microscopy (TEM) analysis sample was prepared using an ion-milling technique (Fig. S4) and the TEM images revealed a distinct *c-a* heterostructure. High-resolution TEM (HRTEM, Fig. 2g) combined with the selected area electron diffraction (SAED) pattern (inset in Fig. 2g) demonstrated that the amorphous matrix contained nanocrystalline Fe_3_B, Ni_4_B_3_, and Co_4_B phases with interplanar spacings of 0.2069, 0.2135, and 0.3678 nm, corresponding to the (002), (202), and (212) facets, respectively. Nanocrystalline domains with sizes of approximately 10–20 nm are generally regarded as the active species for both AOPs and water splitting and presents in large quantities (Fig. S5), corresponding well to the crystalline peaks observed in the XRD pattern. Furthermore, EDS elemental mapping (Fig. 2i) of the yellow square frame region in Fig. 2h confirmed a homogeneous elemental distribution throughout the *c-a* heterostructure and the amorphous base exhibited a uniform elemental distribution (Fig. S6). These observations verified the successful construction of the *c-a* heterostructure, which improved the erosion resistance and increased the density of active sites.

### AOPs Catalytic Performance

**Fig. 3:**
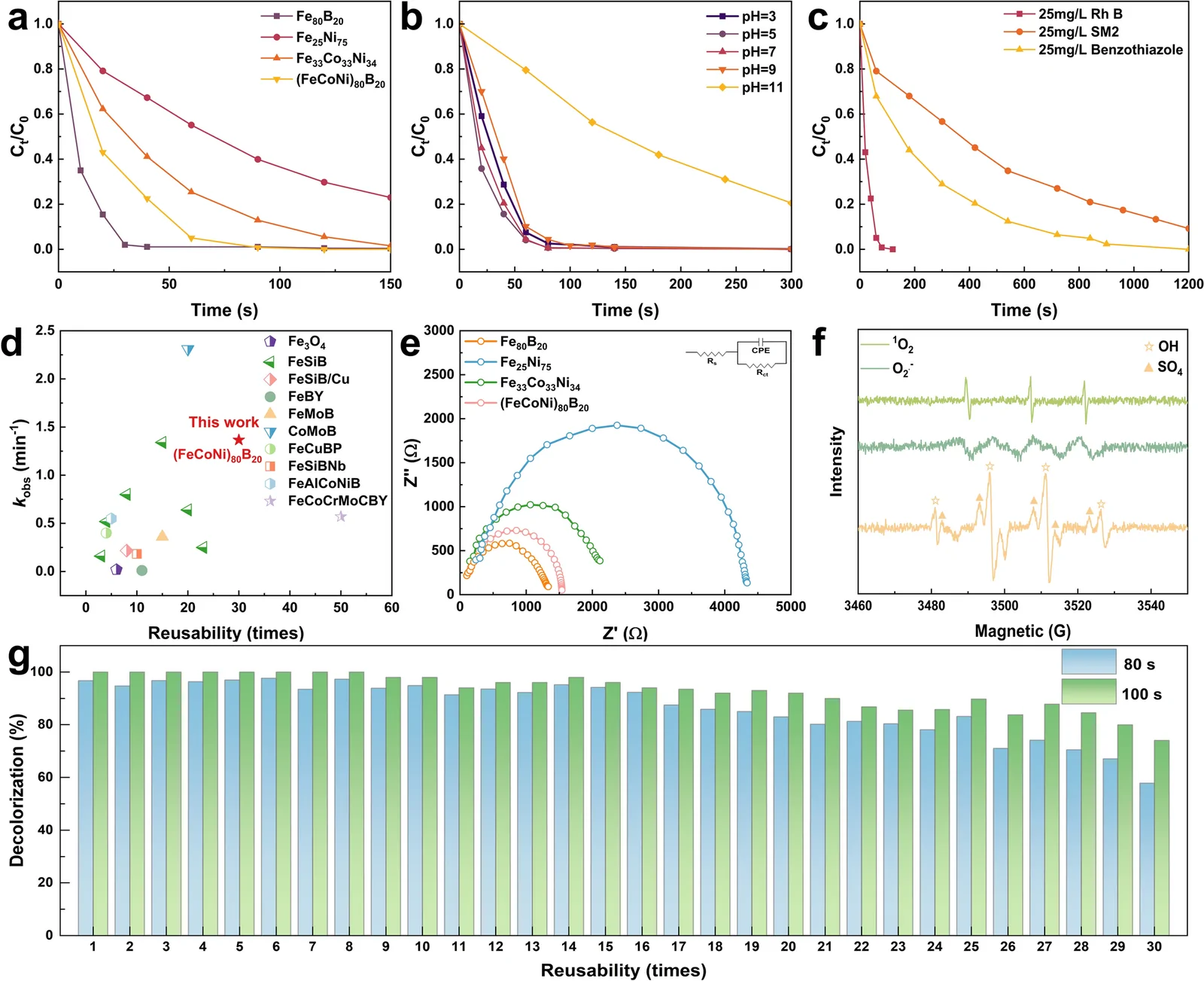
AOP catalytic activity and stability. **a** Degradation efficiency of samples in the presence of 10 mM PS. Degradation efficiency of (FeCoNi)_80_B_20_ fibers **b** under different pH conditions and **c** for various organic pollutants. **d** Comparison of the degradation capacity of (FeCoNi)_80_B_20_ with other reported catalysts, as summarized in Table S1. **e** Nyquist plots of the catalysts measured in 25 mg L^−1^ RhB solution. **f** ESR experiments on (FeCoNi)_80_B_20_. **g** Reusability of (FeCoNi)_80_B_20_ fibers

The catalytic performance of the samples was tested using a 25 mg L^−1^ RhB solution. As shown in Fig. 3a, Fe_80_B_20_ and (FeCoNi)_80_B_20_ achieved nearly complete decolorization within 30 and 90 s, respectively. Because stability is a critical parameter for AOPs, the reusability of Fe_80_B_20_ and (FeCoNi)_80_B_20_ was further examined. The (FeCoNi)_80_B_20_ fibers remained effective for up to 30 reuse cycles, with only a slight decrease in efficiency thereafter (Fig. 3g), whereas Fe_80_B_20_ maintained its activity for 25 cycles (Fig. S7). These results indicated that (FeCoNi)_80_B_20_ exhibited superior AOPs performance by combining high degradation efficiency with enhanced reuse stability (Fig. 3d). To assess the influence of solution pH on degradation behavior, (FeCoNi)_80_B_20_ fibers were tested over a pH range of 3–11 (Fig. 3b). The fastest decolorization was observed at pH 5, exceeding that of conventional Fe-based catalysts. Notably, even at pH 11, more than 80% decolorization was achieved within 300 s, demonstrating pronounced pH adaptability. In addition, (FeCoNi)_80_B_20_ enabled the effective degradation of sulfadimethylpyrimidine (SM2) and benzothiazole, achieving removal efficiencies of 100% and 92% within 1200 s, respectively (Fig. 3c). The effects of environmental parameters, including temperature, catalyst dosage, and RhB concentration, were also investigated (Fig. S8a–c), and the optimal operating conditions were identified as a catalyst loading of 0.5 g L^−1^, an RhB concentration of 25 mg L^−1^, and ambient temperature.

To assess the extent of metal leaching into the contaminant solution, the inductively coupled plasma–optical emission spectroscopy (ICP–OES) was employed after reaction (Fig. S9). During degradation, the leaching of Fe, Co, and Ni was detected, with Fe exhibiting the most pronounced release, indicating that a greater number of Fe sites participated in the catalytic process. This result corresponded the recent researches that Fe played the dominant role as the primary active site in AOPs [75–77]. Electron spin resonance (ESR) spectroscopy confirmed the generation of ·OH, SO4^−^, O_2_^−^, and ^1^O_2_ species during the degradation process (Fig. 3f). The ESR signals revealed abundant ·OH and SO_4_·^−^ radicals, whereas only minor amounts of O_2_^−^ and ^1^O_2_ were detected, highlighting the dominant contribution of radical pathways to pollutant degradation [78, 79]. Furthermore, quenching experiments results of (FeCoNi)_80_B_20_ fibers using furfuryl alcohol (FFA), p-Benzoquinone, methyl alcohol (MeOH), and tertiary butanol (TBA) as quenching agents (Fig. S10). The efficiency exhibited obviously decreased with the addition of the TBA and MeOH that demonstrated that the pivotal role of ·OH and SO4^−^generated radicals in organic molecule degradation. FFA and p-Benzoquinone were used to detect the production of O_2_^−^ and ^1^O_2_ radicals. The results indicated the small amounts of O_2_^−^ and ^1^O_2_ species were produced during the water treatment processes. The results of the quenching experiments were corresponded to the ESR results. Electron transfer characteristics were further examined by electrochemical impedance spectroscopy (EIS) in 25 mg L^−1^ RhB solution (Fig. 3e). The Nyquist plots and fitted equivalent circuits of (FeCoNi)_80_B_20_ exhibited a relatively a lower charge-transfer resistance (R_ct_ = 1443 Ω) than those of Fe_80_B_20_ (R_ct_ = 1259 Ω), Fe_33_Co_33_Ni_34_ (R_ct_ = 2048 Ω), and Fe_75_Ni_25_ (R_ct_ = 4191 Ω), which was consistent with the observed degradation performance. Electrochemical polarization analysis using the Tafel extrapolation method (Fig. S11) revealed that (FeCoNi)_80_B_20_ possessed the lowest self-corrosion current density (I_corr_ = 0.0046 mA cm^−2^), indicating superior corrosion resistance. This enhanced corrosion resistance accounted for the improved reusability and collectively highlights the advantages of the *c-a* heterostructure. To elucidate the origin of the gradual decline efficiency after prolonged cycling, the SEM images of (FeCoNi)_80_B_20_ fibers after 30 degradation cycles were analyzed. The fiber surface exhibited extensive spalling accompanied by precipitate formation (Fig. S12). The corresponding EDS mapping revealed that these precipitates were primarily composed of Fe, C, and O (Fig. S13), which was identified as the main cause of catalyst deactivation.

### Water Splitting Catalytic Performance

**Fig. 4:**
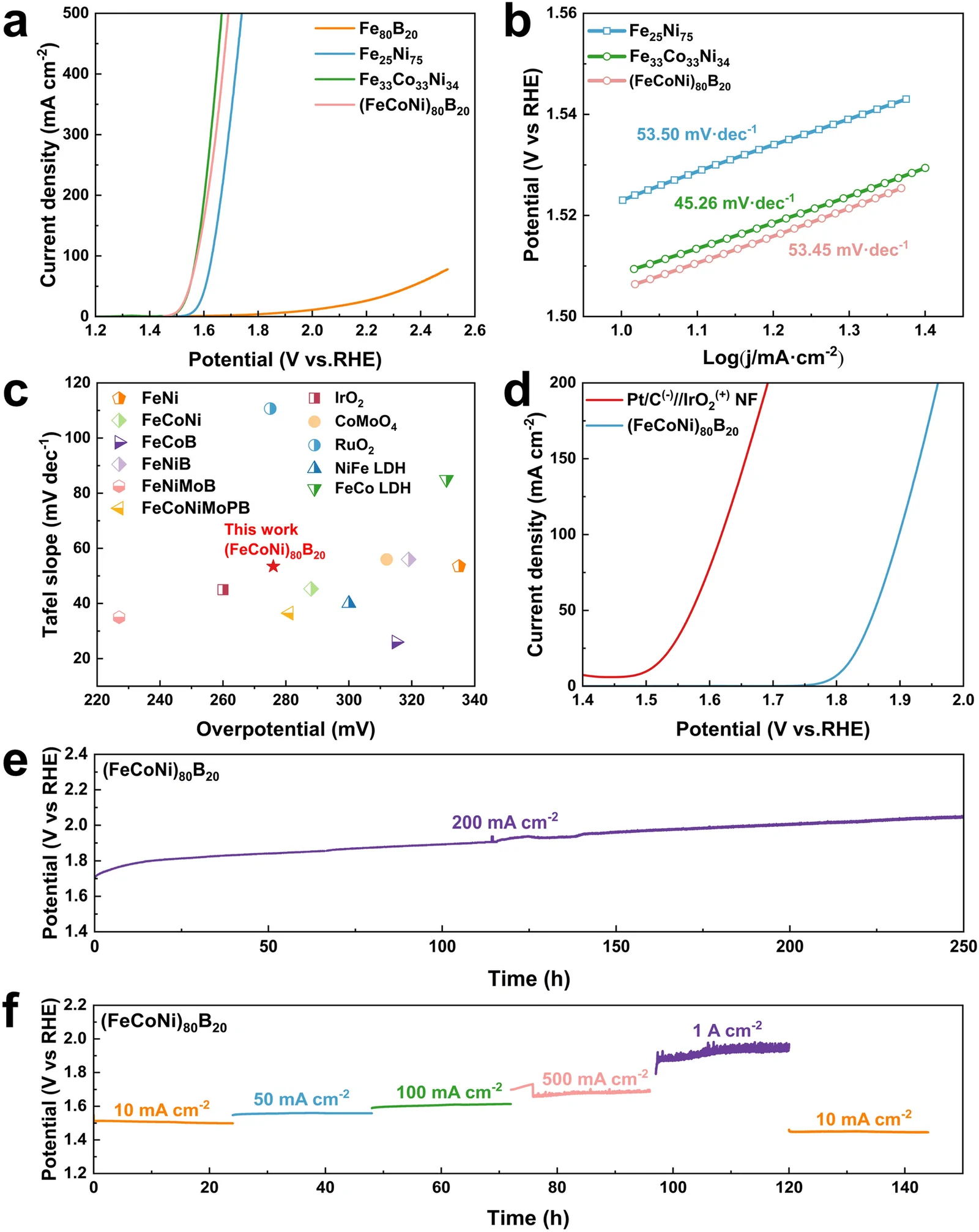
Water splitting catalytic activity and stability. **a** LSV curves for four samples evaluated in 1.0 M KOH (iR corrected, scan rate: 5 mV s^−1^). **b** Corresponding Tafel slopes. **c** Comparison of the overpotential and Tafel slope of (FeCoNi)_80_B_20_ with other reported catalysts, as summarized in Table S2. **d** Comparison of overall water splitting activity of (FeCoNi)_80_B_20_ with Pt/C//IrO_2_ Ni foam electrolyzer. **e** Chronopotentiometry curves of (FeCoNi)_80_B_20_ recorded at a current density of 200 mA cm^−2^. **f** Chronopotentiometry curves of (FeCoNi)_80_B_20_ under practical fluctuating current densities (10, 50, 100, 500, 1000, and 10 mA cm^−2^)

The electrochemical activities of different samples were tested in 1.0 M KOH. For OER, linear sweep voltammetry (LSV) curves illustrate that Fe_33_Co_33_Ni_34_ and (FeCoNi)_80_B_20_ delivered superior catalytic performance, with *ŋ*_10_ values of 288 and 275 mV, respectively (Fig. 4a). In contrast, Fe_80_B_20_ exhibited negligible OER activity and was therefore not considered for further analysis. The (FeCoNi)_80_B_20_ catalyst displayed a Tafel slope of 53.45 mV dec^−1^, comparable to that of Fe_75_Ni_25_ (53.50 mV dec^−1^) and Fe_33_Co_33_Ni_34_ (45.26 mV dec^−1^) (Fig. 4b). As summarized in Fig. 4c, (FeCoNi)_80_B_20_ exhibited competitive OER activity compared with representative catalysts based on transition and noble metals. The EIS measurements yielded Nyquist plots that were fitted using a Randles equivalent circuit (Fig. S14). Among the tested samples, (FeCoNi)_80_B_20_ demonstrated the lowest charge-transfer resistance (R_ct_ ≈ 76.2 Ω), which was markedly lower than those of Fe_80_B_20_ (≈79.4 Ω), Fe_75_Ni_25_ (≈335.0 Ω), and Fe_33_Co_33_Ni_34_ (≈376.4 Ω), indicating the accelerated Faradaic processes [80]. The electrochemical surface area (ECSA) estimated from the double-layer capacitance (C_dl_) obtained by cyclic voltammetry (CV) (Fig. S15) revealed that (FeCoNi)_80_B_20_ possessed the highest C_dl_ value (0.209 mF cm^−2^), significantly exceeding those of Fe_75_Ni_25_ (0.022 mF cm^−2^) and Fe_33_Co_33_Ni_34_ (0.098 mF cm^−2^), suggesting a higher density of exposed active sites. The electrochemical stability was assessed by chronopotentiometry at a current density of 1 A cm^−2^ (Fig. S16), and (FeCoNi)_80_B_20_ maintained stable operation for 120 h with only a modest increase in potential. For HER, the *ŋ*_10_ for Fe_80_B_20_, Fe_75_Ni_25_, Fe_33_Co_33_Ni_34_, and (FeCoNi)_80_B_20_ were 433, 406, 399, and 222 mV, respectively (Fig. S17a). In addition, (FeCoNi)_80_B_20_ exhibited the lowest Tafel slope and R_ct_ among the samples (Fig. S17b, c), demonstrating favorable HER kinetics and enhanced electron transfer capability [81]. The (FeCoNi)_80_B_20_ used as both cathode and anode was further evaluated the overall water splitting performance. As shown in Fig. 4d, the cell voltage of was 1.96 V to reach a current density of 200 mA cm^−2^, which was higher than that of noble-metal-based catalysts but remained competitive with non-precious systems. Notably, the (FeCoNi)_80_B_20_-based electrolyzer operated stably for 250 h at 200 mA cm^−2^ (Fig. 4e) with increased voltage and maintained its durability for 144 h under simulated industrially relevant fluctuating current densities (Fig. 4f). The overall water splitting reaction was conducted in a membrane-free cell, which led to local pH shifts that would increase the kinetic overpotential of the electrode reactions [82]. Thus, the cell voltage increased before reaching stability. The ICP-OES results (Table S3) exhibited significant leaching of the active elements element after 250 h stability test at 200 mA cm^−2^ that may contribute to the observed activity degradation. At the same time, the fiber surface remained smooth and self-supporting (Fig. S18), with uniformly distributed surface elements (Fig. S19). The TEM characterization was preformed to furtherly explore the reasons for the sharp decline in stability. The HRTEM image revealed a substantial reduction in nanocrystal size after prolonged stability testing (Fig. S20). This is another reason for the limited stability because the nanocrystalline exhibited better activity for water splitting [65, 83].

### Water Splitting Catalytic Performance in Reclaimed Water

**Fig. 5:**
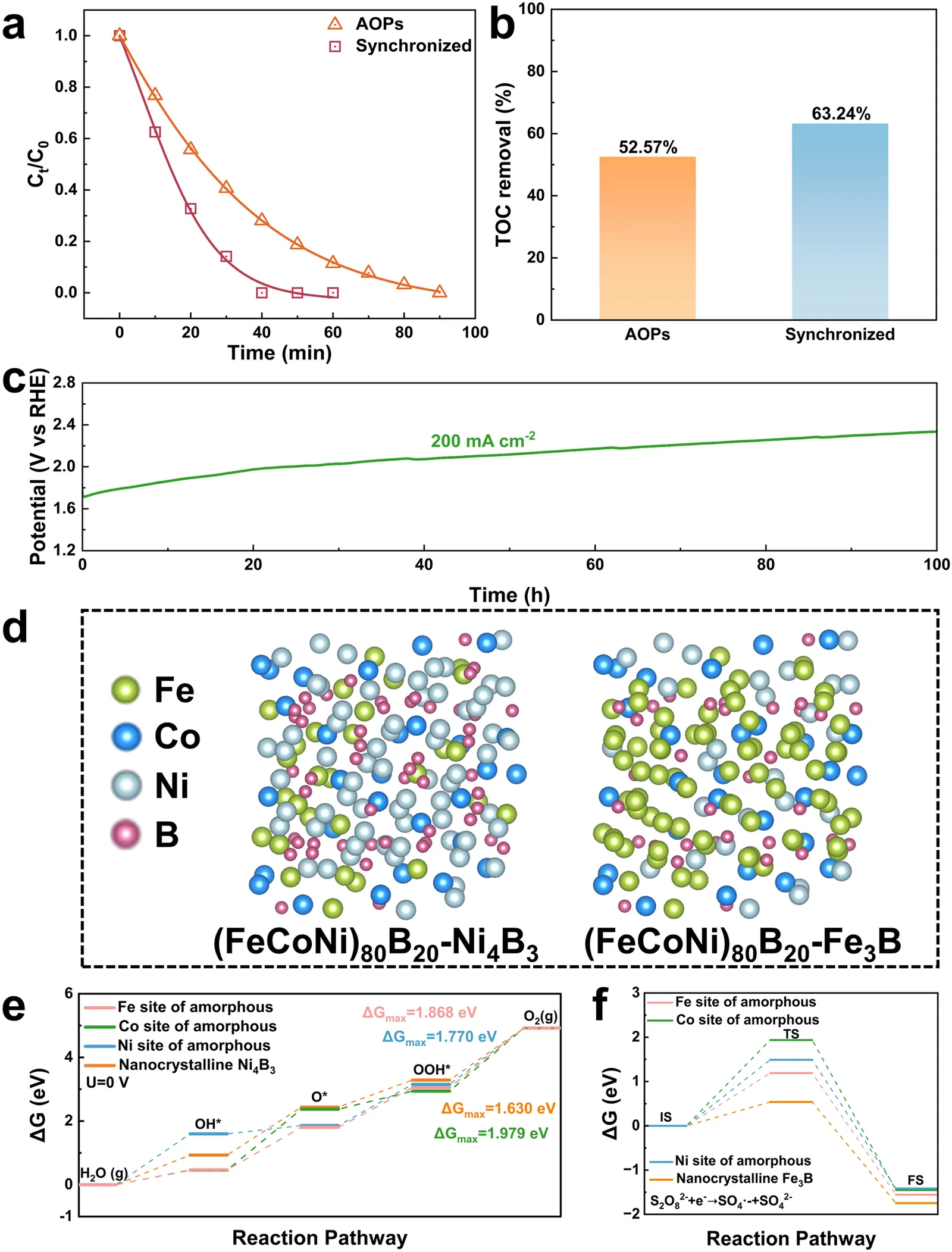
Synchronized catalytic activity and stability of the integrated AOPs–water splitting process. **a** Degradation efficiency of (FeCoNi)_80_B_20_ in simulated reclaimed water (1.0 M KOH + 25 mg L^−1^ RhB) under simultaneous overall water splitting at a current density of 200 mA cm^−2^. **b** Comparison of TOC removal rates between AOP-only process and annealed synchronized process. **c** Chronopotentiometry curve of the AEM electrolyzer operated at a current density of 200 mA cm^−2^ in simulated reclaimed water (without iR correction). **d** Calculation models of *c-a* for (FeCoNi)_80_B_20_-Ni_4_B_3_ and (FeCoNi)_80_B_20_-Fe_3_B. **e** Gibbs free energy changes for the four steps in the OER process at Fe, Co, and Ni site of the amorphous (FeCoNi)_80_B_20_ and nanocrystalline Ni_4_B_3_. **f** Calculated energy profile for S_2_O_8_^2−^

Based on the AOPs and water splitting catalytic properties of (FeCoNi)_80_B_20_, these two processes can be conducted simultaneously. Overall water splitting was performed at a current density of 200 mA cm^−2^ in simulated reclaimed water (1.0 M KOH with 25 mg L^−1^ RhB). Complete decolorization of the solution was achieved within 60 min, whereas the water splitting process maintained stable operation for approximately 115 h (Figs. 5a and S21a). The total organic carbon (TOC) removal rate reached 63.24% (Fig. 5b). Compared with conventional decolorization, the synchronized process exhibited higher degradation efficiency. The presence of RhB and Na_2_S_2_O_8_ exerted only a negligible influence on OER performance (Fig. S22a, b), whereas the decolorization efficiency was significantly accelerated under an applied current owing to enhanced electron transfer (Fig. S22c) [84]. The ICP–OES analysis revealed the leaching behavior of Fe, Co, and Ni after the two processes (Table S4). The higher Fe leaching rate observed in the synchronized process was attributed to the more rapid oxidation of Fe^0^ during water splitting, which promoted the degradation reaction. The XPS spectra collected after AOPs, water splitting, and synchronized process exhibited no substantial changes in peak features, indicating the structural stability of (FeCoNi)_80_B_20_ under different operating conditions (Fig. S23a–c). Notably, the Fe 2*p* (Fig. S24a) and Ni 2*p* (Fig. S24b) spectra displayed the evident negative shifts after the synchronized process, suggesting an increased electron density around Fe and Ni atoms, which is beneficial for catalytic activity [85]. Furthermore, the synchronized process resulted in the highest proportions of M–OH and M–OOH species (Fig. S23c), implying the generation of a greater number of active sites. Raman spectroscopy was employed to probe the variations of active species in the surface during different catalytic processes (Fig. S25). Compared with the other samples, the fiber after the synchronized process exhibited enhanced Raman bands at 281, 472, 554, and 715 cm^−1^, which were characteristic of FeOOH [86] and NiOOH [15], indicating the formation of the active M-OOH species during operation. These results were consistent with the XPS results.

To assess practical applicability, an AEM electrolyzer was assembled using (FeCoNi)_80_B_20_ as both anode and cathode in simulated reclaimed water (Fig. S26). The AEM electrolyzer sustained a current density of 200 mA cm^−2^ for approximately 100 h (Fig. 5c). Compared with recent amorphous alloy researches, the (FeCoNi)_80_B_20_ maintained electrocatalytic activity for water splitting in reclaimed water while simultaneously achieving efficient reclaimed water purification (Table S5). The XRD patterns were tested to further demonstrate the stability (Fig. S27). The crystalline peaks became less sharp after the synchronized process, suggesting the occurrence of structural reconstruction during the process. The LSV curves before and after the 100 h stability tests were implied to confirm the stability (Fig. S28). The overpotential increased 17 mV at 10 mA cm^−2^ that demonstrated the great stability in the synchronized process. In contrast, when operated after decolorization alone, (FeCoNi)_80_B_20_ remained stable for only approximately 75 h at 200 mA cm^−2^, which was significantly shorter than that observed in the synchronized process (Fig. S21b). The SEM images obtained after 115 h of synchronized operation demonstrated that the fibers retained their self-supporting structure, while abundant surface precipitates were observed (Fig. S29a). EDS mapping confirmed that these precipitates were primarily composed of Fe, C, and O (Fig. S29b). Compared with fibers after decolorization, no pronounced surface flaking was detected, which may contribute to the improved stability during subsequent water splitting operations. The combined catalytic activity and durability demonstrated in the synchronized process highlights the feasibility of this strategy for industrial applications.

### Mechanism Investigation for Bifunctional Catalyst

The adsorption behavior of OH* was examined by leading methanol into the electrolyte (Fig. S30a–c). The difference in current density induced by the methanol oxidation reaction (MOR) can be used as an indicator of the extent of surface OH* accumulation [87]. Stronger OH* binding favors a higher decolorization rate, whereas moderate binding strength is more conducive to efficient water splitting. Owing to its moderate OH* adsorption energy, (FeCoNi)_80_B_20_ facilitated the generation of ·OH at the anode during the synchronized process, thereby enhancing the degradation efficiency. The process in which applied voltage drives the single‑electron oxidation of water to generate highly oxidative •OH is referred to as EOPs. Meanwhile, because an oxidant was added to the simulated wastewater, AOPs can also occur in the absence of applied voltage. This behavior indicated a coupled reaction pathway in which AOPs and EOPs proceeded simultaneously, resulting in accelerated degradation kinetics [57].

Because Fe and Ni sites serve as the primary active centers for AOPs and overall water splitting, respectively, the structural models of *c-a* (FeCoNi)_80_B_20_-Ni_4_B_3_ and (FeCoNi)_80_B_20_-Fe_3_B were constructed based on TEM observations (Fig. 5d). According to the Gibbs free energy changes (ΔG) calculated for the OER four-electron pathway on amorphous (FeCoNi)_80_B_20_ and nanocrystalline Ni_4_B_3_ for OER 4e^−^ pathway (Fig. S31), the rate-determining step on the Fe, Co, and Ni site at 0 V corresponded to the oxidation of *OOH to O_2_, with ∆G_max_ values of 1.868, 1.979, 1.770, and 1.630 eV, respectively (Fig. 5e). Compared with the amorphous substrate, nanocrystalline Ni_4_B_3_ exhibited a markedly lower ∆G_max_ with an increased OH desorption energy, which accounted for its superior catalytic activity toward both OER and AOPs. To elucidate the activation pathway of S_2_O_8_^2−^, transition-state simulations were performed for amorphous (FeCoNi)_80_B_20_ and nanocrystalline Fe_3_B (Fig. S32). The nanocrystalline Fe_3_B displayed a substantially lowest energy barrier (0.539 eV) (Fig. 5f) and the Fe site exhibited the lower barrier (1.191 eV) at the amorphous phase that is consistent with the results of degradation experiments. In addition, the final state of the interfacial activation process exhibited relative energies of − 2.749 and − 2.285 eV for amorphous (FeCoNi)_80_B_20_ and nanocrystalline Fe_3_B, respectively, indicating that the heterolytic cleavage of S_2_O_8_^2−^ occurred more readily on nanocrystalline Fe_3_B. Compared with the amorphous matrix, the nanocrystalline domains exhibited enhanced activity for both processes, highlighting the advantage of the crystalline–amorphous heterostructure. The total density of states (TDOS) analysis of amorphous (FeCoNi)_80_B_20_, (FeCoNi)_80_B_20_-Ni_4_B_3_, and (FeCoNi)_80_B_20_-Fe_3_B revealed that the Ni sites increased the density of empty states near the Fermi level, indicating the presence of abundant unpaired electrons associated with Ni (Fig. S33a–c) [88]. Partial density of states (pDOS) analysis further indicated that Fe, Co, and Ni sites collectively regulated the adsorption characteristics of (FeCoNi)_80_B_20_, thereby facilitating intermediate desorption in both catalytic reactions. The partial radial distribution functions (PRDFs) of Fe, Co, and Ni in (FeCoNi)_80_B_20_ are shown in Fig. S34. The lower PRDF peak intensities corresponded to lower coordination numbers, which favored the generation of additional active sites [89]. The analysis of the first PRDF peak reveals the specific coordination number for each sample [90]. Notably, the Fe–Ni pair exhibited the lowest peak intensity, suggesting that the interaction between Fe and Ni sites plays an important role in improving the activity of both AOPs and water splitting. Overall, the DFT results demonstrated that Fe and Ni sites were intrinsically beneficial for the two catalytic processes, while the *c-a* heterostructure increased the number of active sites, and the nanocrystalline domains further enhanced the catalytic activity.

## Conclusion

In this study, a bifunctional catalyst integrating AOPs and water splitting was developed and demonstrated to operate effectively in reclaimed water. The synergistic interaction among multiple elements and the stable amorphous structure of MEAA enabled the integration of both reaction pathways, while the modulation of oxygen intermediate binding strength achieved a balance between the two functionalities. As a result, the (FeCoNi)_80_B_20_ catalyst exhibited outstanding catalytic activity and durability for both AOPs and water splitting. In addition, accelerated dye degradation was observed under water splitting conditions, further highlighting the cooperative effect between the two processes. When configured as both anode and cathode in an anion-exchange-membrane electrolyzer operating in reclaimed water, the medium-entropy metallic glass electrodes sustained a current density of 200 mA cm^−2^ at approximately 1.96 V for more than 100 h, with only a moderate increase in cell voltage over time. Although challenges associated with electrolyzer corrosion in reclaimed water remain, the low cost and high catalytic performance of this bifunctional system underscore the feasibility of water electrolysis in reclaimed water and highlight its potential for industrial applications.

## Supplementary Information

Below is the link to the electronic supplementary material.

Supplementary file1 (DOCX 7609 kb)
