# Supplementary material for: Integrating Overall Water Splitting with Advanced Oxidation for Wastewater Treatment Using a Bifunctional Medium-Entropy Amorphous Alloy
Source: Nanomicro Lett. 2026 Apr 16;18:333. doi: 10.1007/s40820-026-02172-1 (PMC13083759; doi:10.1007/s40820-026-02172-1)
Supplement: Supplementary file 1 — Supplementary file1 (DOCX 7609 kb) [file 40820_2026_2172_MOESM1_ESM.docx]

Supporting Information for

**Integrating Overall Water Splitting with Advanced Oxidation for Wastewater Treatment Using a Bifunctional Medium-Entropy Amorphous Alloy**

Yifan Cui^1,2,3,4^, Yonghui Wang^5^, Bo Li^1,4^, Jiaqi Huang^1,4^, Le Bo^1,4^, Hengqi Liu^5^, Mahlanyane Kenneth Mathe^6^, Murodjon Samadiy^7^, Shengfeng Guo^8^, Hongxian Shen^1,4^, Jianfei Sun^1,4^, Sida Jiang^1,2,3,4*^

^1^School of Materials Science and Engineering, Harbin Institute of Technology, Harbin 150001, P. R. China

^2^Laboratory for Space Environment and Physical Sciences, Harbin Institute of Technology, Harbin 150001, P. R. China

^3^Frontier Science Center for Interaction between Space Environment and Matter, and National Key Laboratory of Space Environment and Matter Behaviors

^4^National Key Laboratory for Precision Hot Forming, Harbin Institute of Technology, Harbin 150001, P. R. China

^5^School of Physics, Harbin Institute of Technology, Harbin 150001, P. R. China

^6^Department of Chemistry, University of South Africa, Johannesburg 1709, South Africa

^7^Department of Chemical Engineering and Biotechnology, Karshi State Technical University, Karshi 180100, Uzbekistan

^8^School of Materials and Energy, Chongqing Key Laboratory for Advanced Materials and Technologies of Clean Energies, Southwest University, Chongqing 400715, P. R. China

*Corresponding author. E-mail: [jiangsida@hit.edu.cn](mailto:jiangsida@hit.edu.cn) (Sida Jiang)

**Supplementary Figures and Tables**


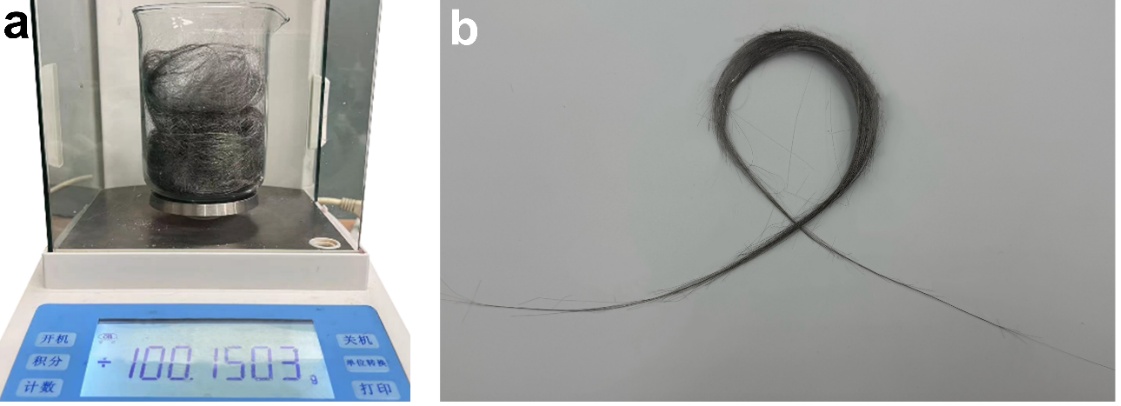


**Fig. S1** **a** One manufacturing batch resulted in around ~ 100 g of (FeCoNi)_80_B_20_ fibers. **b** The digital photograph of the fiber prepared by melt-extraction


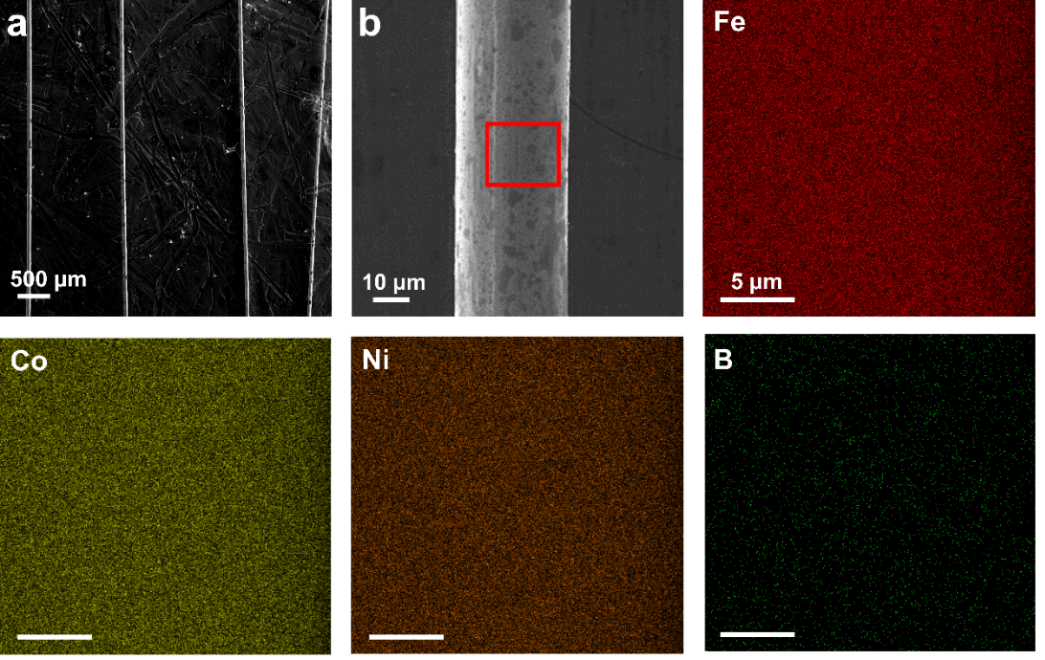


# **Fig. S2 a** SEM images of the fibers with smooth surface that prepared by melt-extraction. **b** EDS maps of the (FeCoNi)_80_B_20_ as-prepared fiber surface. The SEM images demonstrated the fibers have a diameter of approximately 30 μm with uniform surface elemental distribution

**
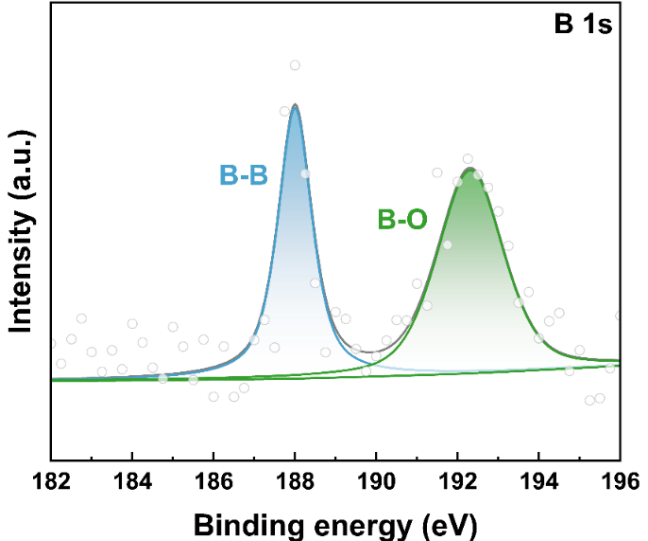
**

# **Fig. S3** B 1*s* XPS spectra of as-prepared (FeCoNi)_80_B_20_ fiber. The B 1*s* spectrum confirmed the existence of B-B and B-O bonds


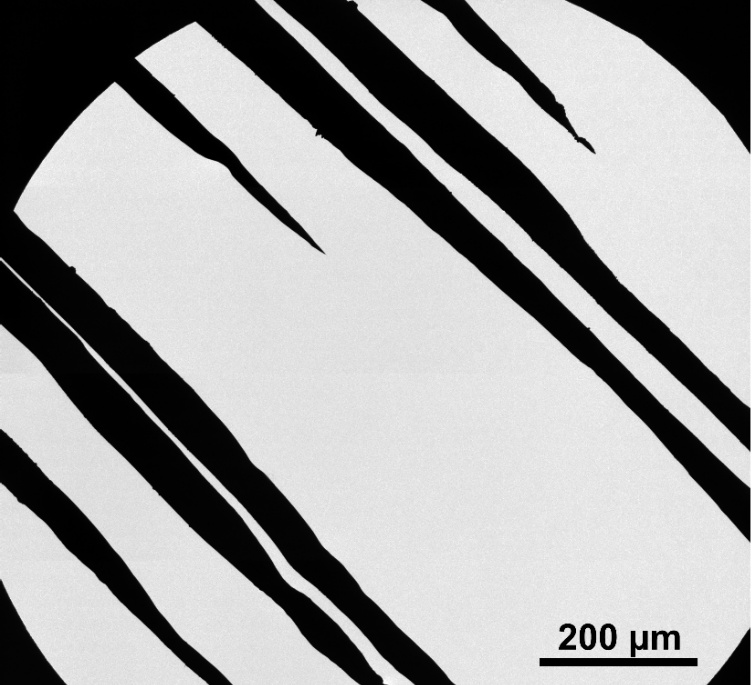


# **Fig. S4** TEM image of the (FeCoNi)_80_B_20_ fibers after ion milling. TEM analysis was performed on a thin region of the sample

#
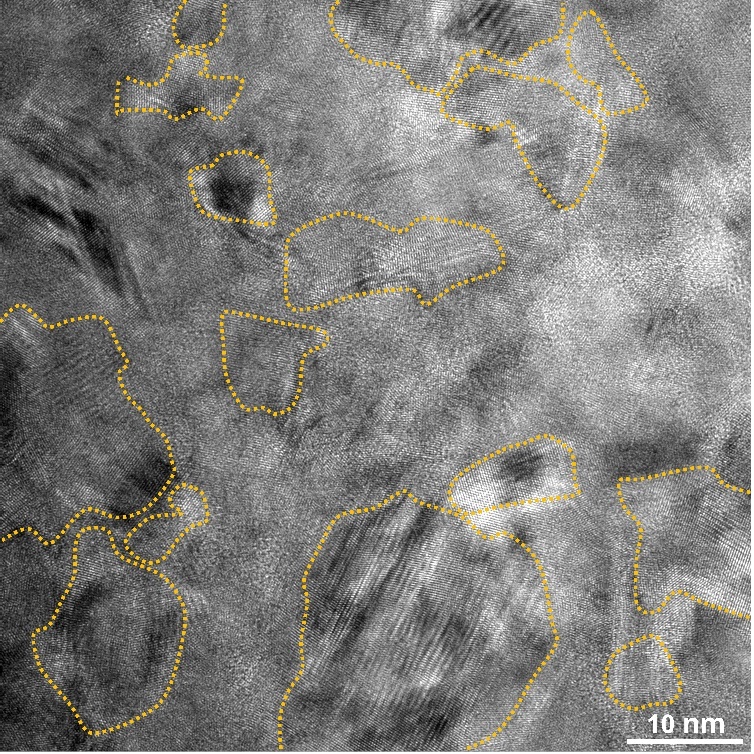


# **Fig. S5** HRTEM image of the (FeCoNi)_80_B_20_ fiber that demonstrated the construction of the crystalline–amorphous (*c-a*) heterostructure. The nanocrystals, with diameters ranging from 10–20 nm and present in large quantities


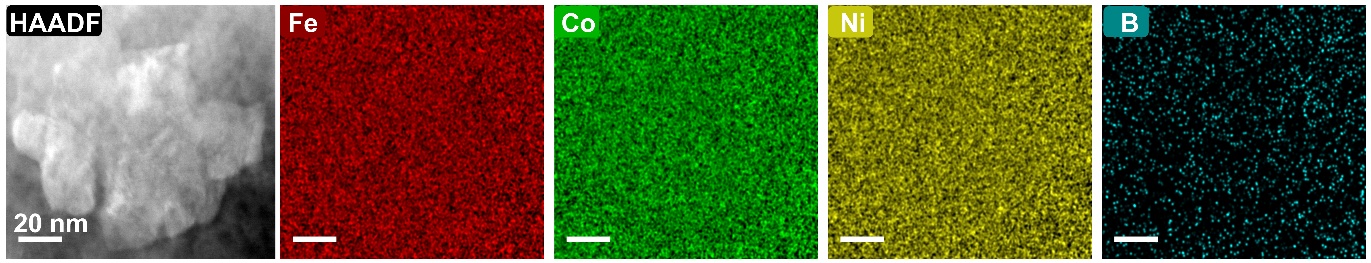


**Fig. S6** EDS maps of (FeCoNi)_80_B_20_ fiber that exhibited a uniform elemental distribution of amorphous


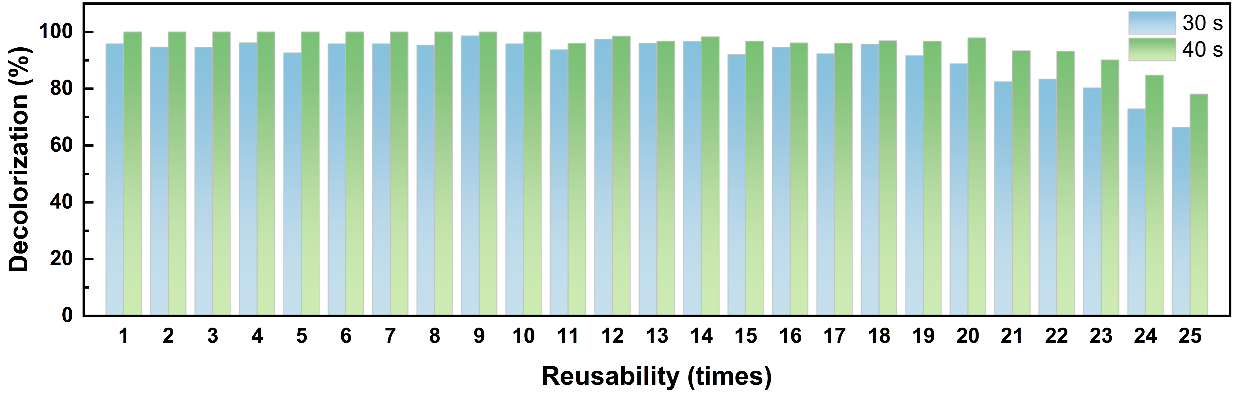


# **Fig. S7** Reusability of Fe_80_B_20_ fibers. The degradation time was set at 30 and 40 seconds to evaluate the catalyst's reusability. The Fe_80_B_20_ fibers maintained its activity for 25 cycles


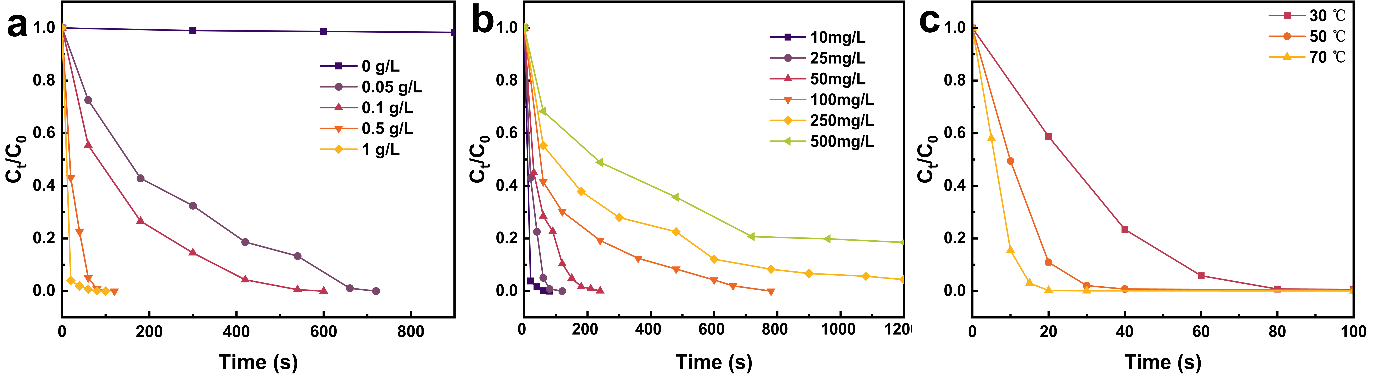


**Fig. S8** Degradation efficiency of (FeCoNi)_80_B_20_ fibers for various **a** fiber dosage, **b** contaminant concentration and (c) temperature. The optimal operating conditions were identified as a catalyst loading of 0.5 g L^−1^, an RhB concentration of 25 mg L^−1^, and ambient temperature


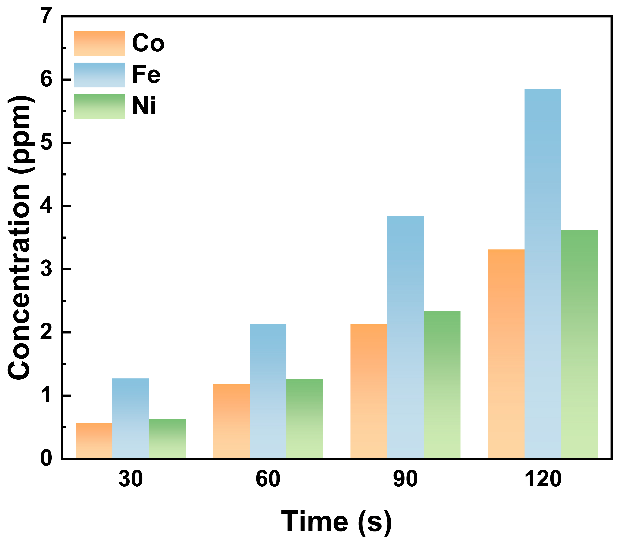


# **Fig. S9** ICP-OES results of Fe, Co and Ni elements during the degradation process. During degradation, the leaching of Fe, Co, and Ni was detected, with Fe exhibiting the most pronounced release, indicating its dominant role as the primary active site in AOPs


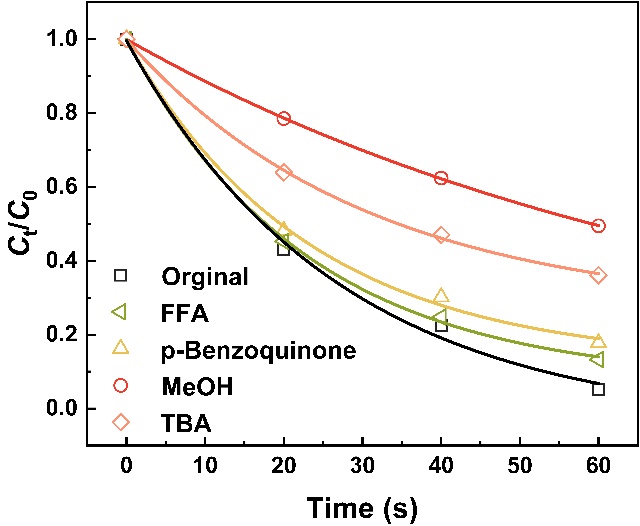


**Fig. S10** Quenching experiments results of (FeCoNi)_80_B_20_ fibers using furfuryl alcohol (FFA), p-Benzoquinone, methyl alcohol (MeOH) and tertiary butanol (TBA) as quenching agents. The results demonstrated that the pivotal role of ·OH and SO4·^−^generated radicals in organic molecule degradation and the small amounts of O_2_·^−^, and ^1^O_2_ species were produced during the water treatment processes


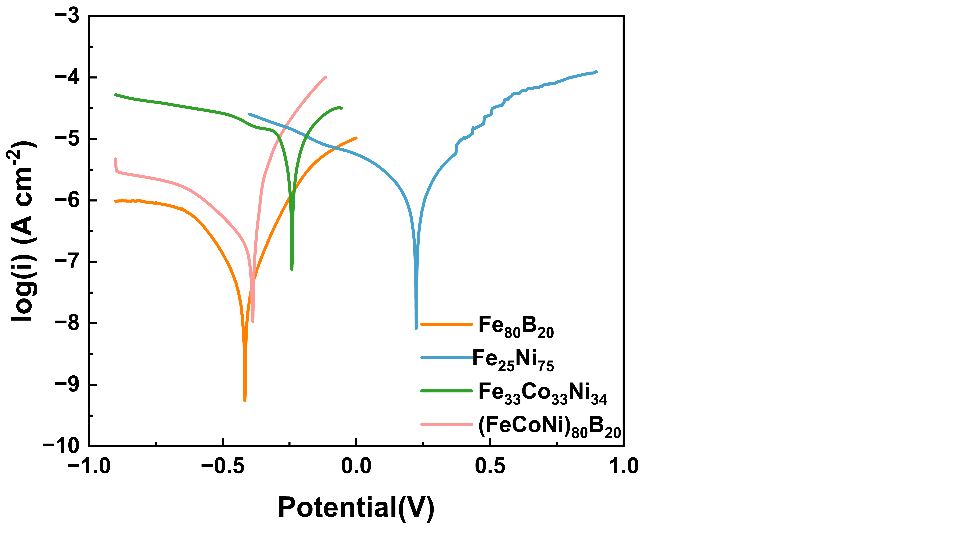


# **Fig. S11** Electrochemical polarization curves of Fe_80_B_20_, Fe_75_Ni_25_, Fe_33_Co_33_Ni_34_ and (FeCoNi)_80_B_20_ fibers. Electrochemical polarization analysis using the Tafel extrapolation method revealed that (FeCoNi)_80_B_20_ possessed the lowest self-corrosion current density (I_corr_ = 0.0046 mA cm^−2^), indicating superior corrosion resistance

**
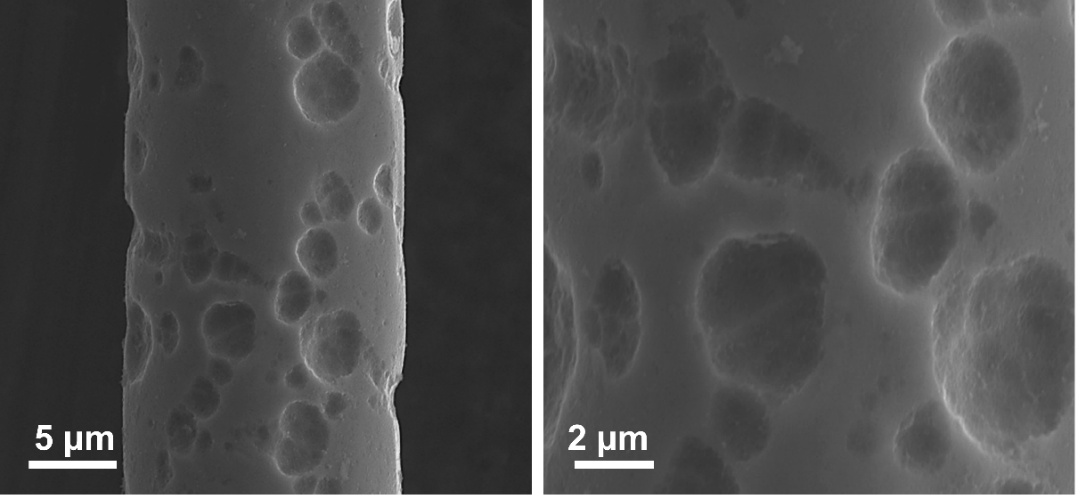
**

# **Fig. S12** SEM images of the (FeCoNi)_80_B_20_ fiber after 30 times cycle. The fiber surface exhibited extensive spalling accompanied by precipitate formation

**
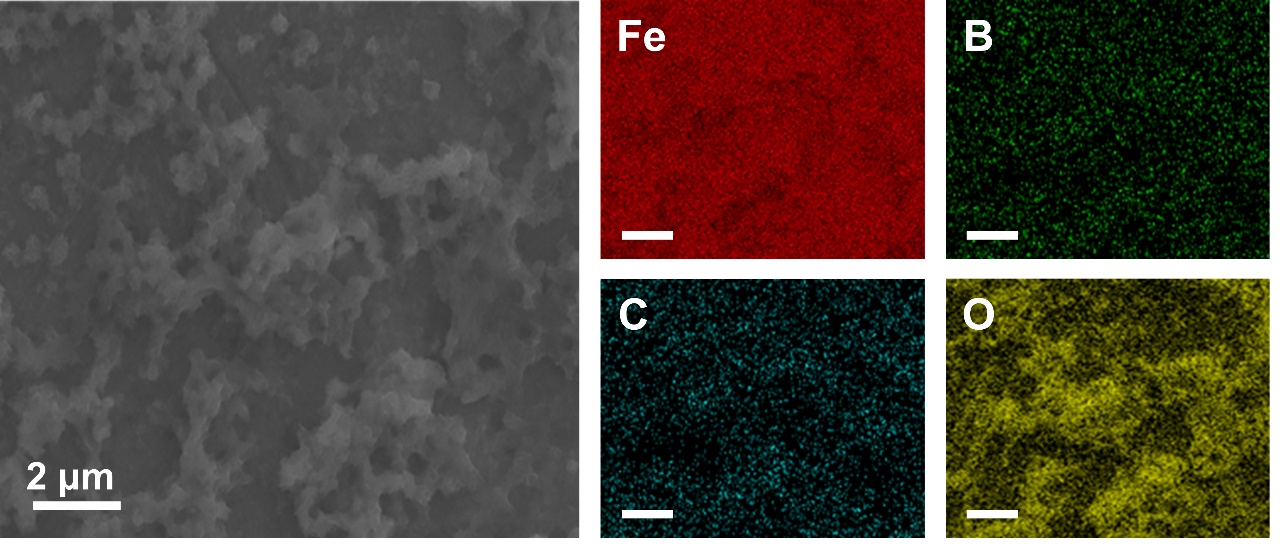
**

**Fig. S13** EDS maps of the precipitates of (FeCoNi)_80_B_20_ fiber after 30 times cycle. The EDS mapping revealed that these precipitates were primarily composed of Fe, C, and O, which was identified as the main cause of catalyst deactivation

**
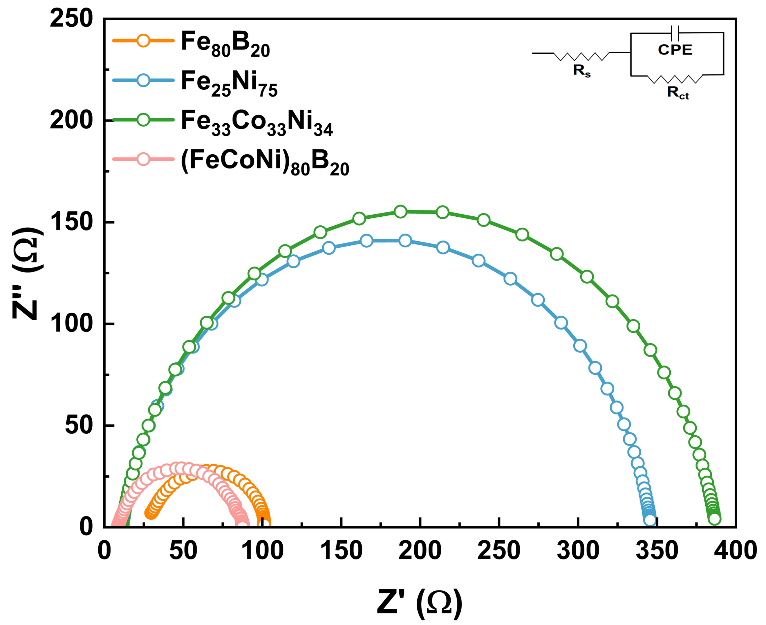
**

# **Fig. S14** Nyquist plots of the catalysts in 1.0 M KOH for OER. Among the tested samples, (FeCoNi)_80_B_20_ demonstrated the lowest charge-transfer resistance, indicating the accelerated Faradaic processes.

**
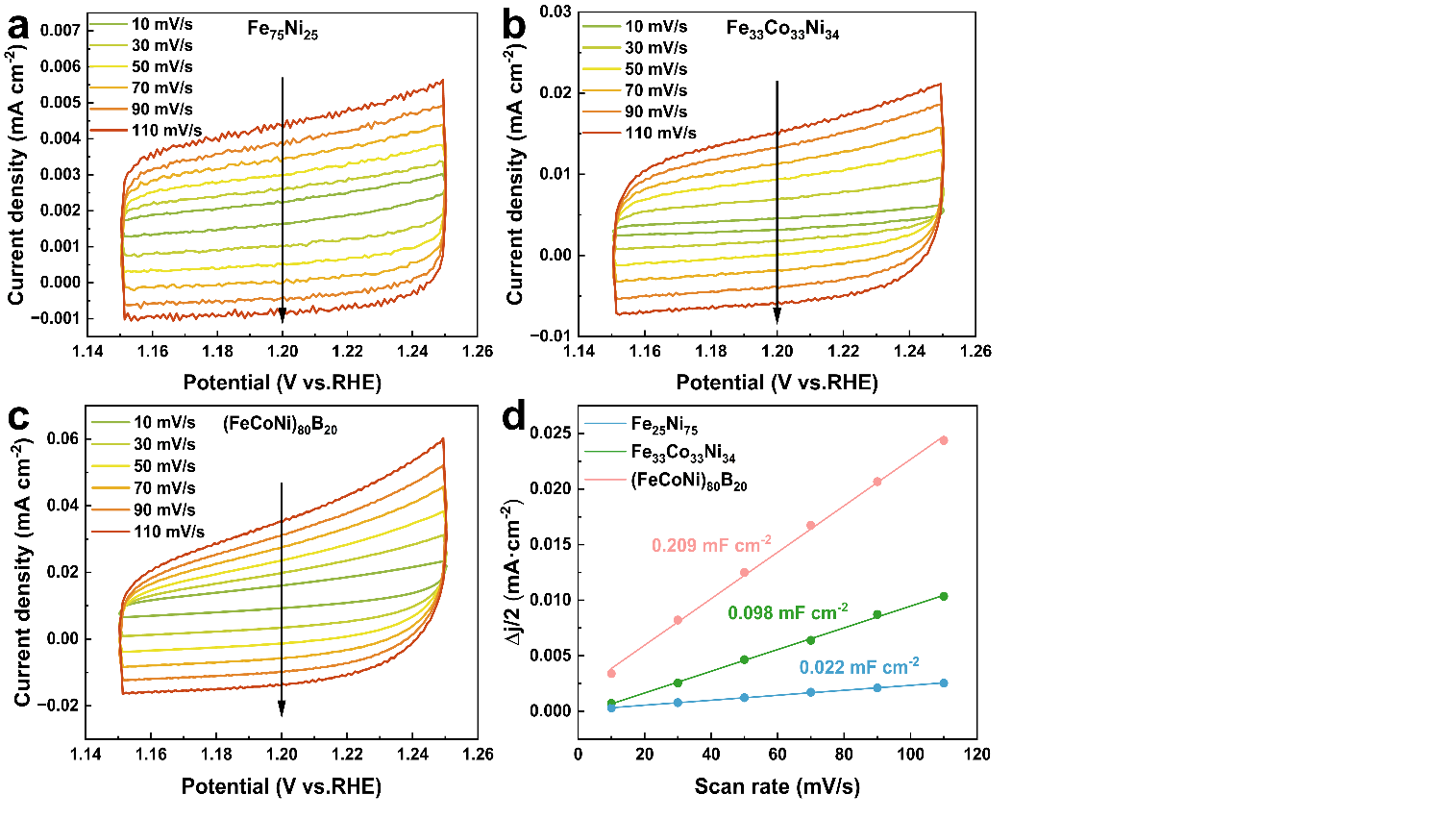
**

# **Fig. S15** CV curves of the fiber in 1 M KOH at different scan rates (10, 30, 50, 70, 90, 110 mV s^-1^): **a** Fe_75_Ni_25_, **b** Fe_33_Co_33_Ni_34_, and **c** (FeCoNi)_80_B_20_ fibers. **d** C_dl_ corresponding to different scan rates. According to the reported typical value, the specific capacitance (C_s_) in 1 M KOH is selected as C_s_ = 0.040 mF cm^2^. Therefore, the (FeCoNi)_80_B_20_ fibers exhibit the maximum ECSA value, indicating abundant active sites, which correspond to optimal OER performance

#
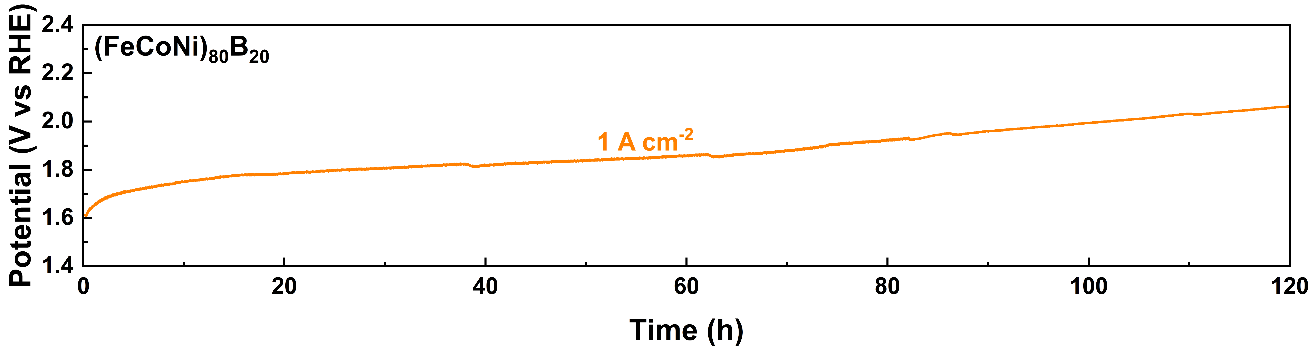


# **Fig. S16** Chronopotentiometry curves of (FeCoNi)_80_B_20_ under current density of 200 mA cm^-2^ without iR correction. The (FeCoNi)_80_B_20_ fiber maintained stable operation for 120 h with only a modest increase in potential


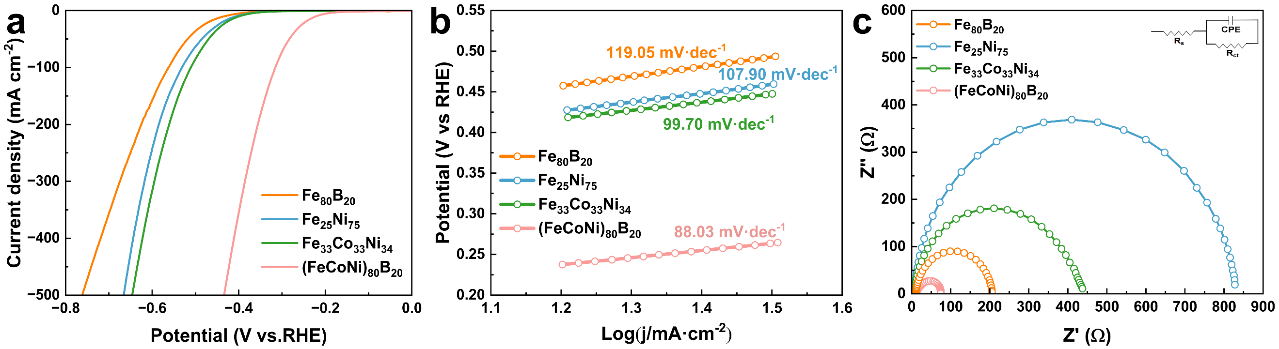


# **Fig. S17 Electrocatalytic HER performance.** **a** LSV curves of different samples (iR corrected, scanning rate: 5 mV s^-1^) in 1.0 M KOH. **b** Tafel plots for the data presented in a. **c** Nyquist plots, where the inset shows the equivalent circuit model. The (FeCoNi)_80_B_20_ electrode revealed the lowest overpotential and R*_ct_*

_
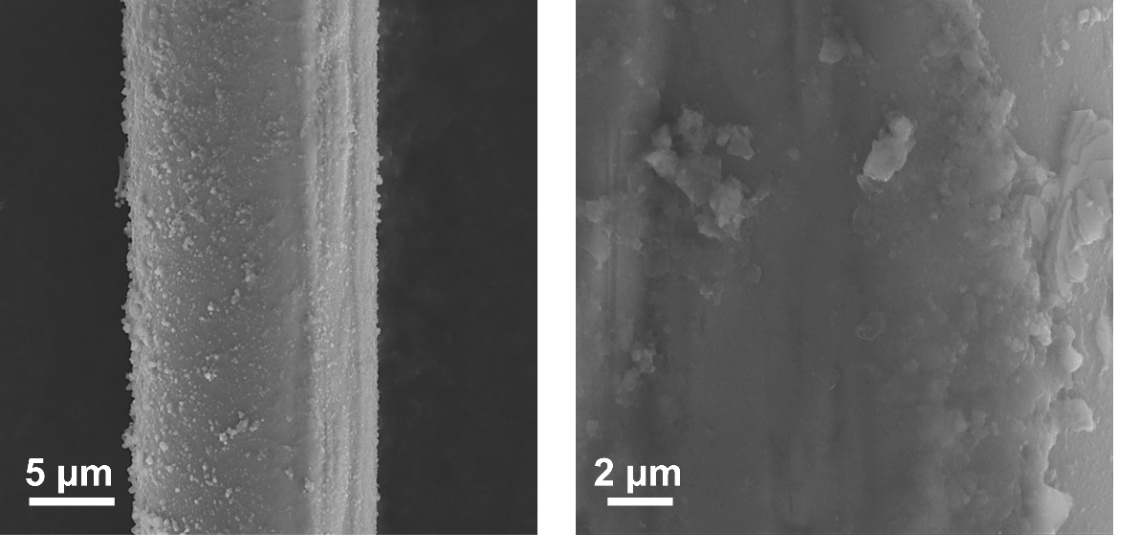
_

# **Fig. S18** SEM images of the (FeCoNi)_80_B_20_ fiber after after 250 h water splitting stability test. After 250 h of stability testing, the fiber surface remained smooth and self-supporting

**
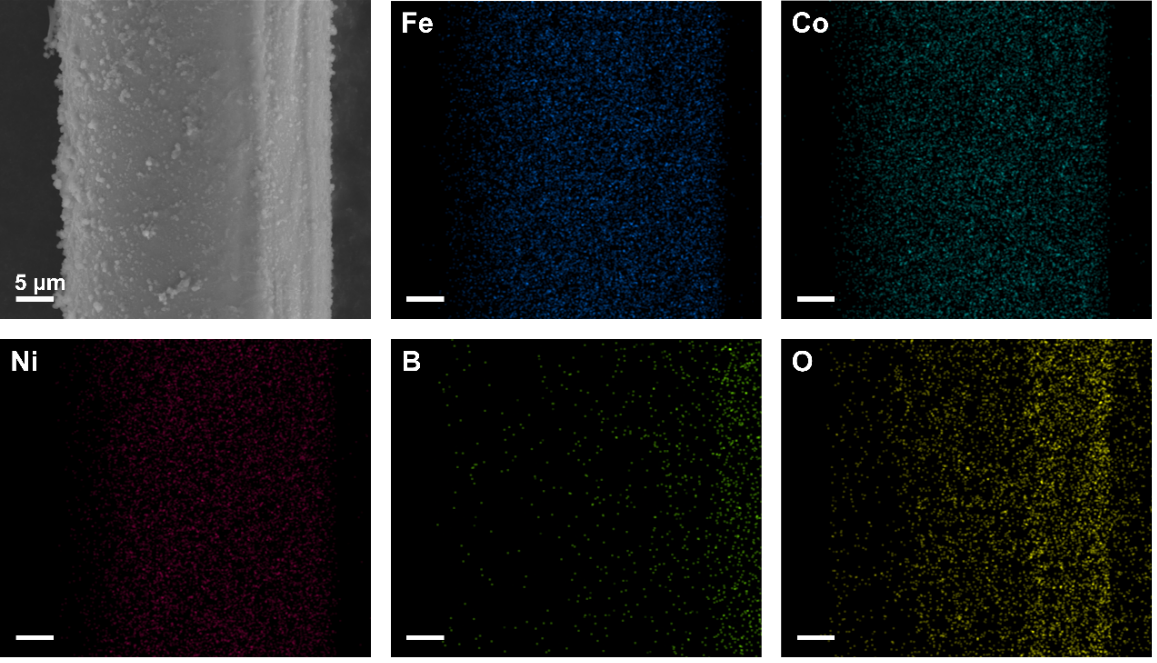
**

# **Fig. S19** EDS maps of the (FeCoNi)_80_B_20_ fiber after 250 h water splitting stability test. The surface of the fiber maintained uniform elemental distribution that was obviously different with the fiber after AOPs reusability test

# **
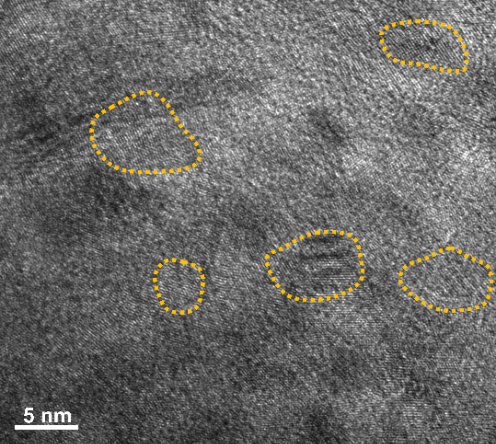
**

# **Fig. S20** HRTEM image of the (FeCoNi)_80_B_20_ fiber after 250 h water splitting stability test. The decrease in nanocrystal size likely contributes to the reduced OER activity

**
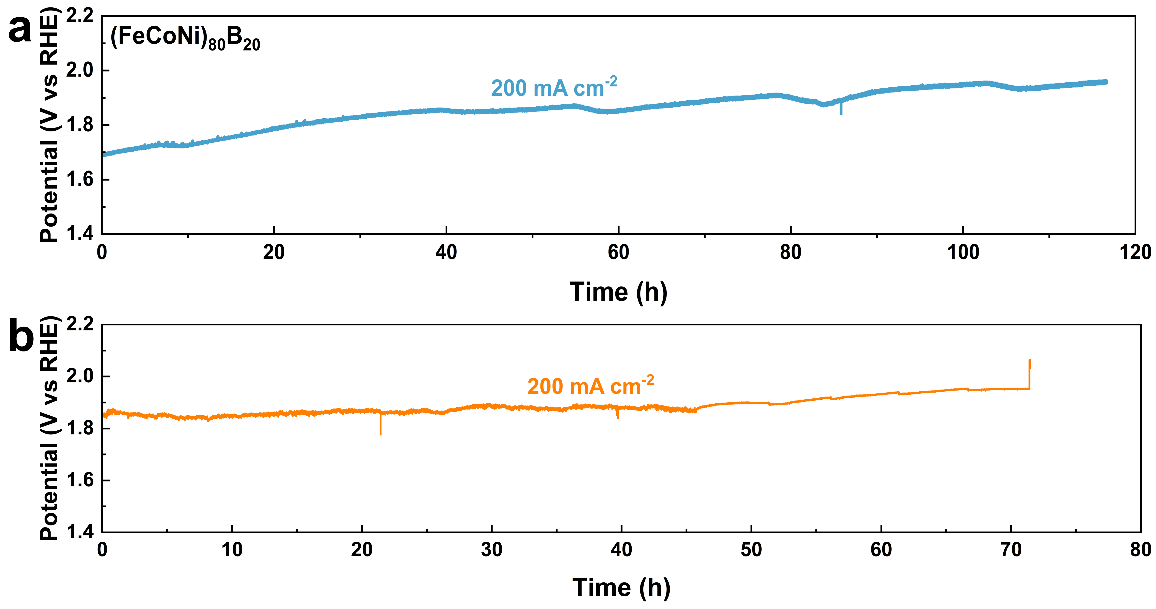
**

# **Fig. S21 a** Chronopotentiometry curves of (FeCoNi)_80_B_20_ at 200 mA cm^-2^ of the synchronized process without iR correction. **b** Chronopotentiometry curves of (FeCoNi)_80_B_20_ at 200 mA cm^-2^ after degradation without iR correction. The fiber shown more excellent stable during the synchronized process

**
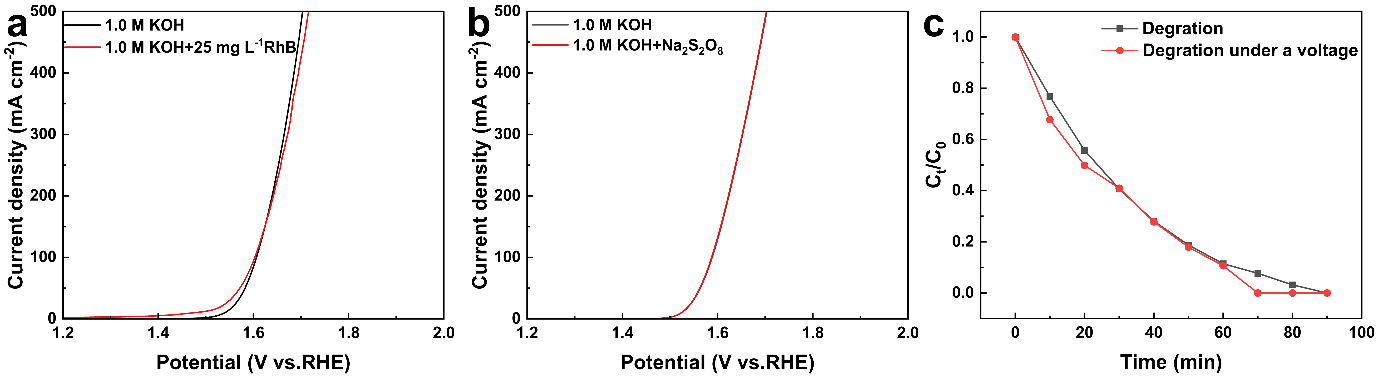
**

# **Fig. S22** The LSV curves of the addition of **a** RhB and **b** Na_2_S_2_O_8_. **c** Degradation efficiency comparison on the change of applied current. The presence of RhB and Na_2_S_2_O_8_ exerted only a negligible influence on OER performance, whereas the decolorization efficiency was significantly accelerated under an applied current owing to enhanced electron transfer

**
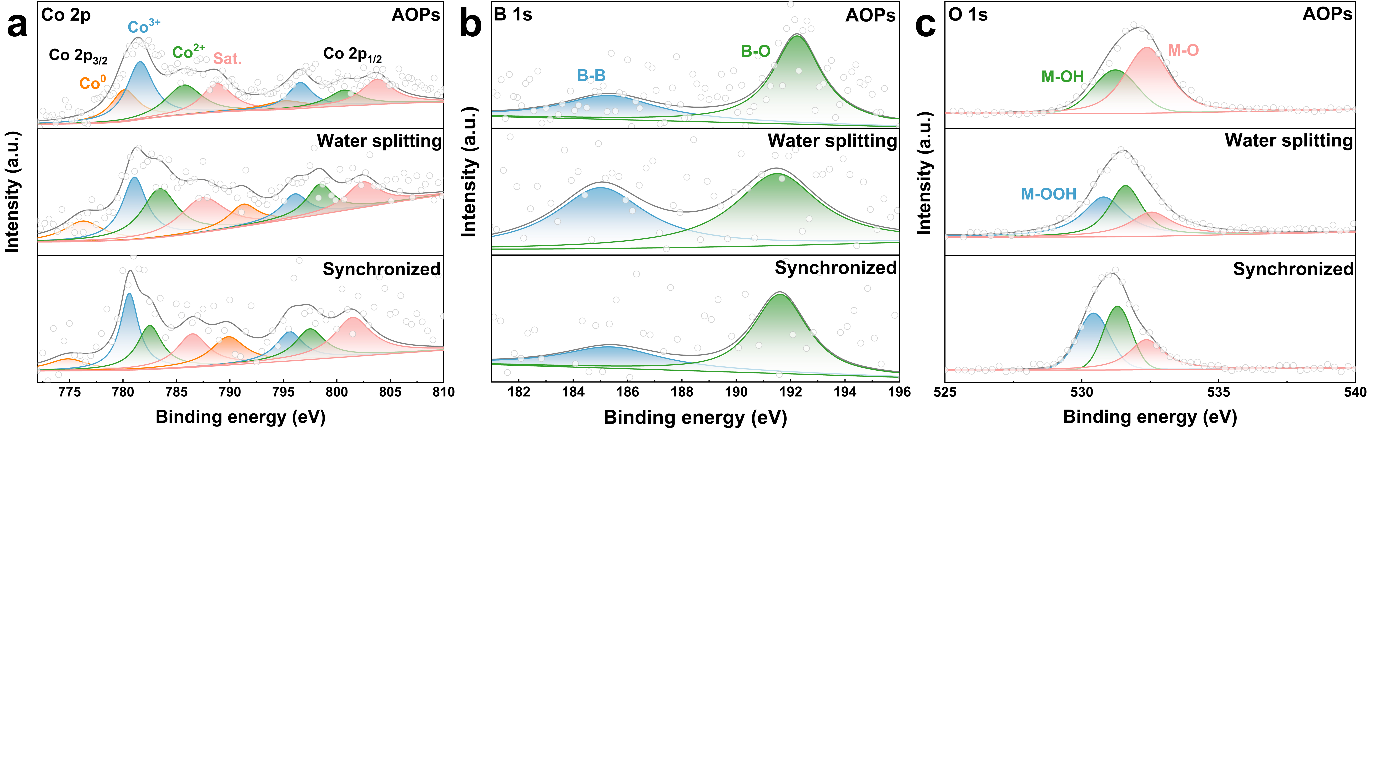
**

# **Fig. S23 a** Co 2*p*, **b** B 1*s* and **c** O 1*s* XPS spectra of (FeCoNi)_80_B_20_ during the AOPs, water splitting and synchronized process. The XPS spectra exhibited no substantial changes in peak features, indicating the structural stability of (FeCoNi)_80_B_20_ under different operating conditions


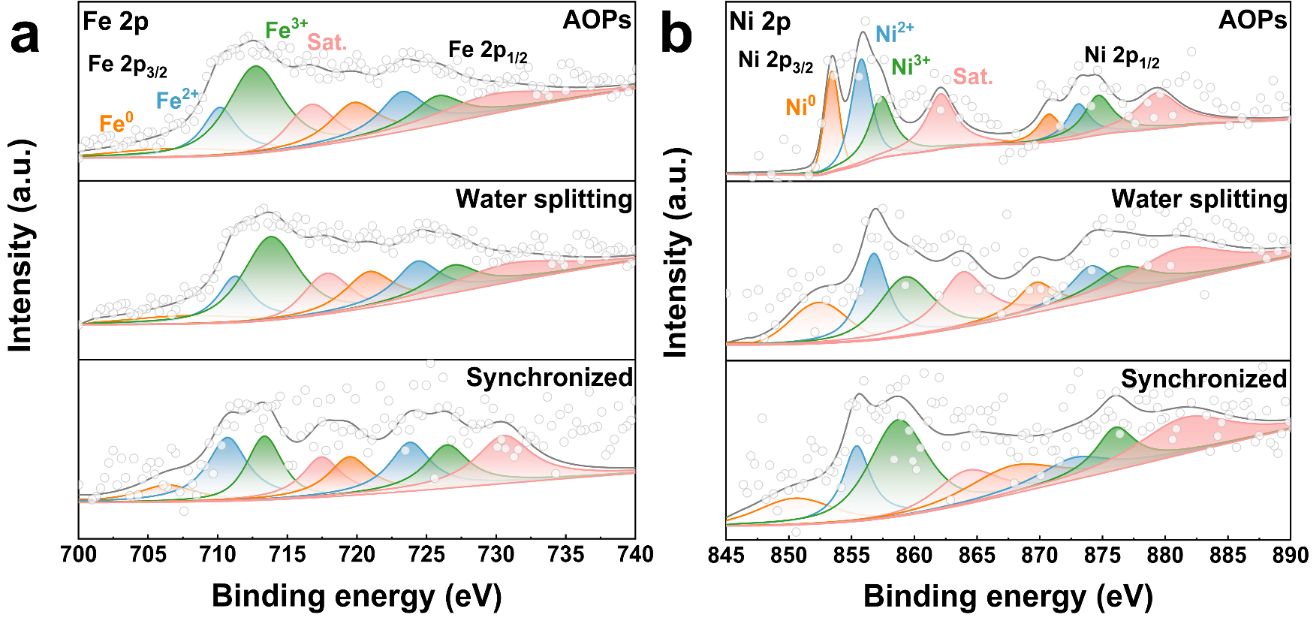


# **Fig. S24 a** Fe *2p* and **b** Ni 2*p* XPS spectra of (FeCoNi)_80_B_20_ during the AOPs, water splitting and synchronized process. The Fe 2*p* and Ni 2*p* spectra displayed the evident negative shifts after the synchronized process, suggesting an increased electron density around Fe and Ni atoms, which is beneficial for catalytic activity

**
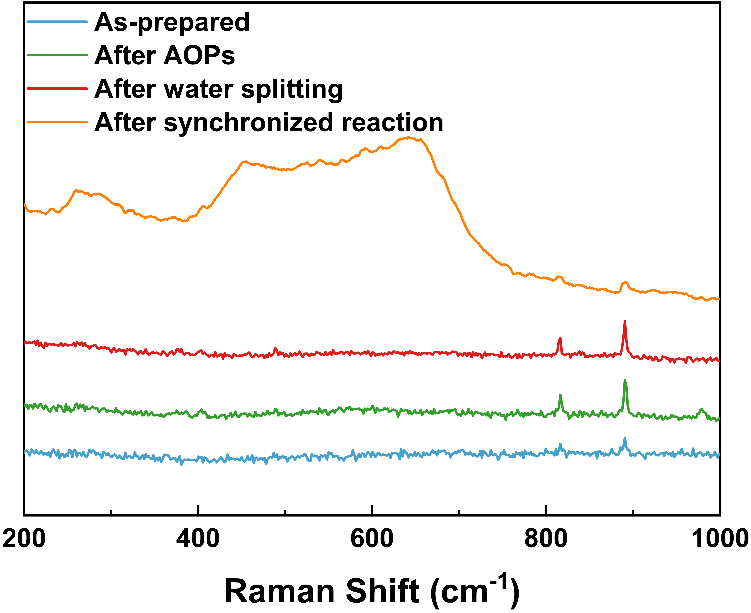
**

**Fig. S25** Raman spectroscopy of (FeCoNi)_80_B_20_ after AOPs, water splitting and synchronized processes compared with the as-prepared fiber. The fiber after the synchronized process exhibited enhanced Raman bands at 281, 472, 554, and 715 cm^−1^, which were characteristic of FeOOH and NiOOH, indicating the formation of the active M-OOH species during operation

**_
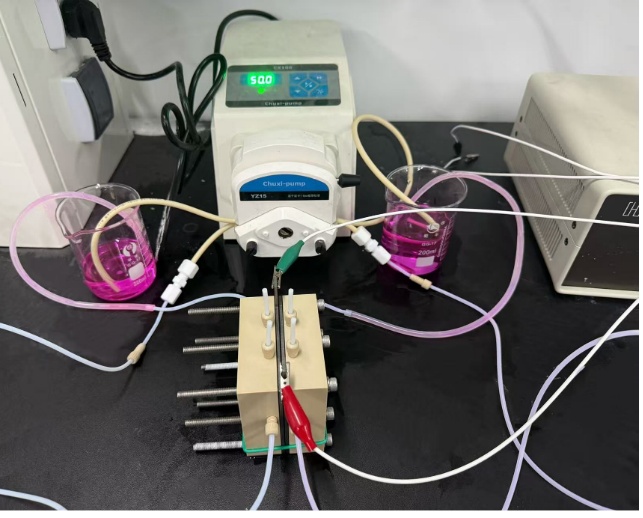
_**

**Fig. S26** The digital photograph of the AEM system of the synchronized process. A peristaltic pump was used to regulate the flow of 1.0 M KOH electrolyte to both sides of the electrolyser at a rate of 2.5 mL min^-1^ throughout the testing


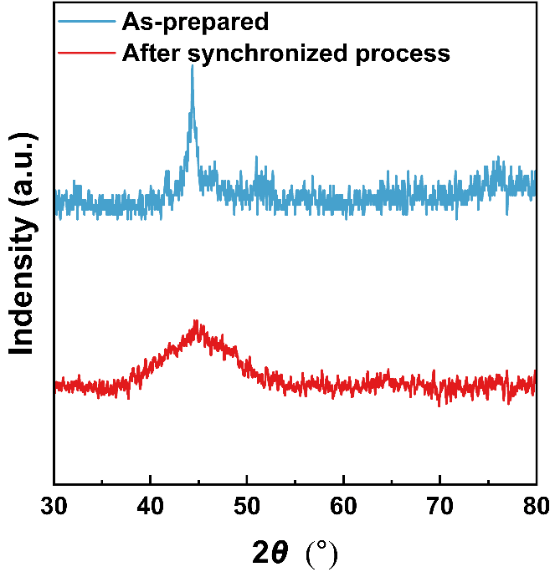


**Fig. S27** X-ray diffraction patterns of (FeCoNi)_80_B_20_ before and after the synchronized process. The crystalline peaks became less sharp after the synchronized process, suggesting the occurrence of structural reconstruction during the process

**_
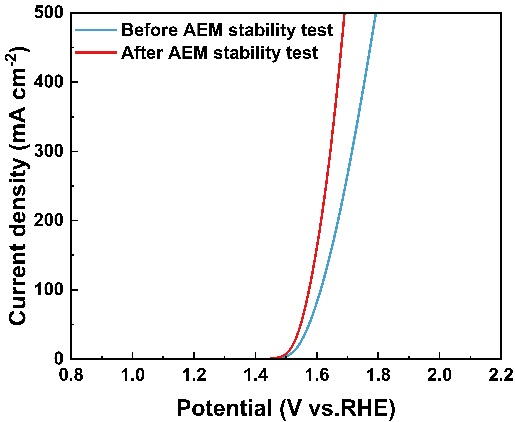
_**

# **Fig. S28** LSV curves of (FeCoNi)_80_B_20_ before and after 100 stability tests at current density of 200 mA cm^-2^ in simulated reclaimed water (iR corrected, scanning rate: 5 mV s^-1^)

**_
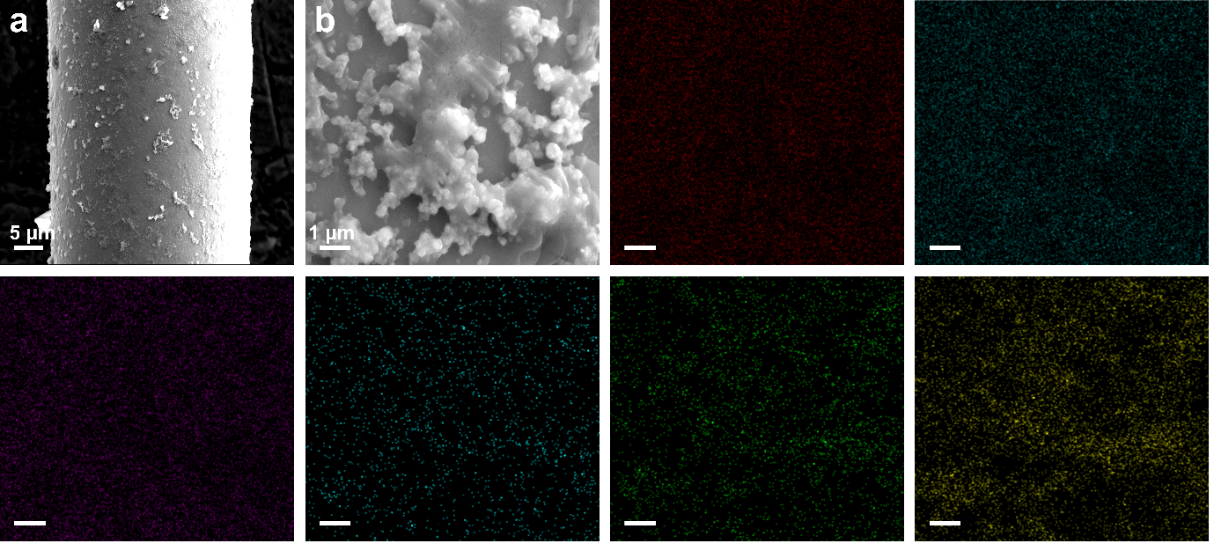
_**

# **Fig. S29** (a) SEM image and (b) EDS maps of the (FeCoNi)_80_B_20_ after 115 h synchronized process. The SEM images demonstrated that the fibers retained their self-supporting structure, while abundant surface precipitates were observed. EDS mapping confirmed that these precipitates were primarily composed of Fe, C, and O

**
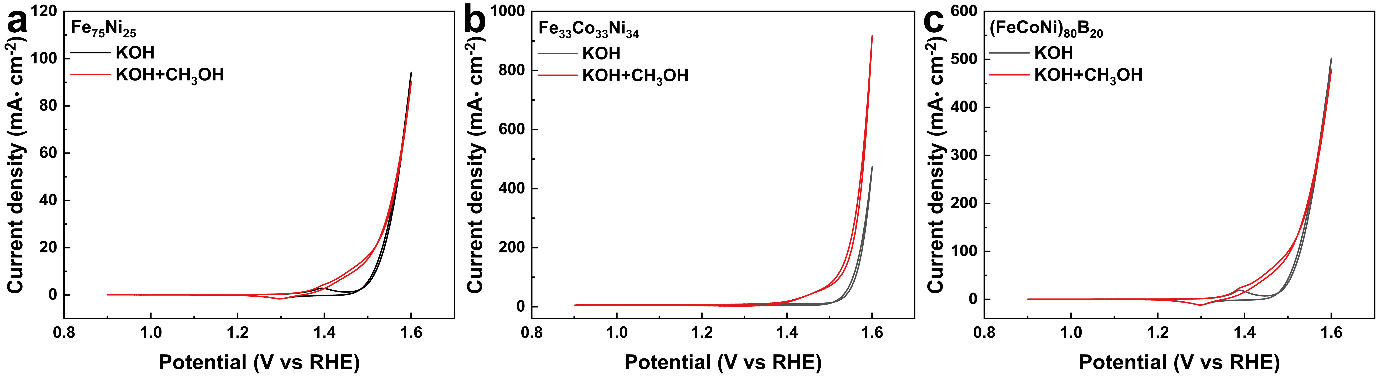
**

# **Fig. S30** Cyclic voltammetry (CV) curves of (a) Fe_75_Ni_25_, (b) Fe_33_CO_33_Ni_34_ and (c) (FeCoNi)_80_B_20_ in 1 M KOH with and without methanol (1 M). Scan rate: 50 mV/s. The (FeCoNi)_80_B_20_ exhibited the moderate adsorption energy of the OH*


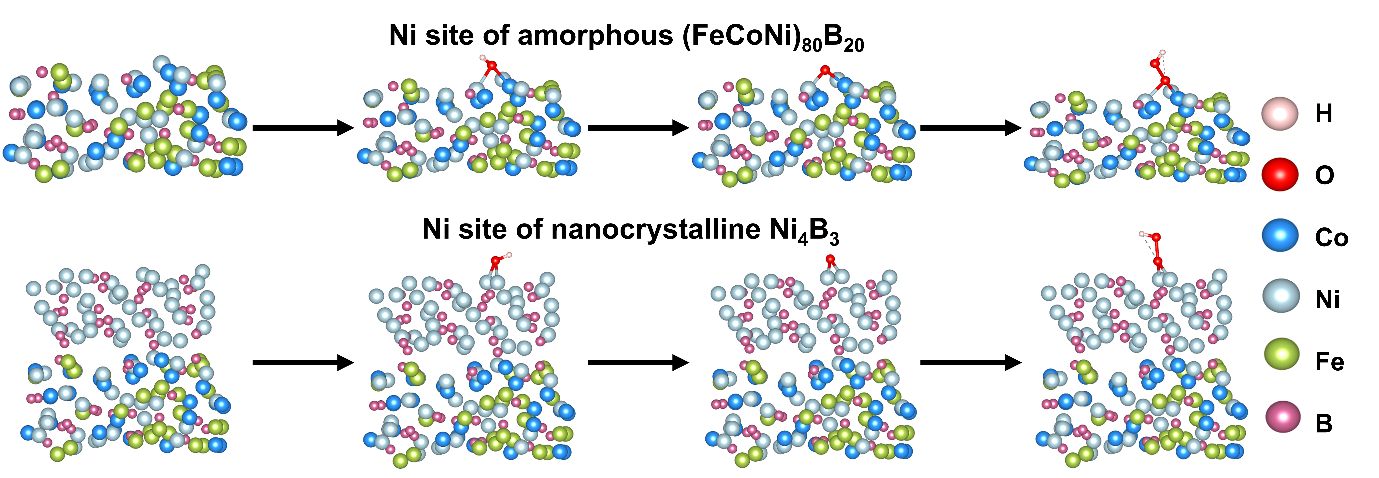


# **Fig. S31** The surface adsorption process of OER for Ni site of amorphous (FeCoNi)_80_B_20_ and nanocrystalline Ni_4_B_3_


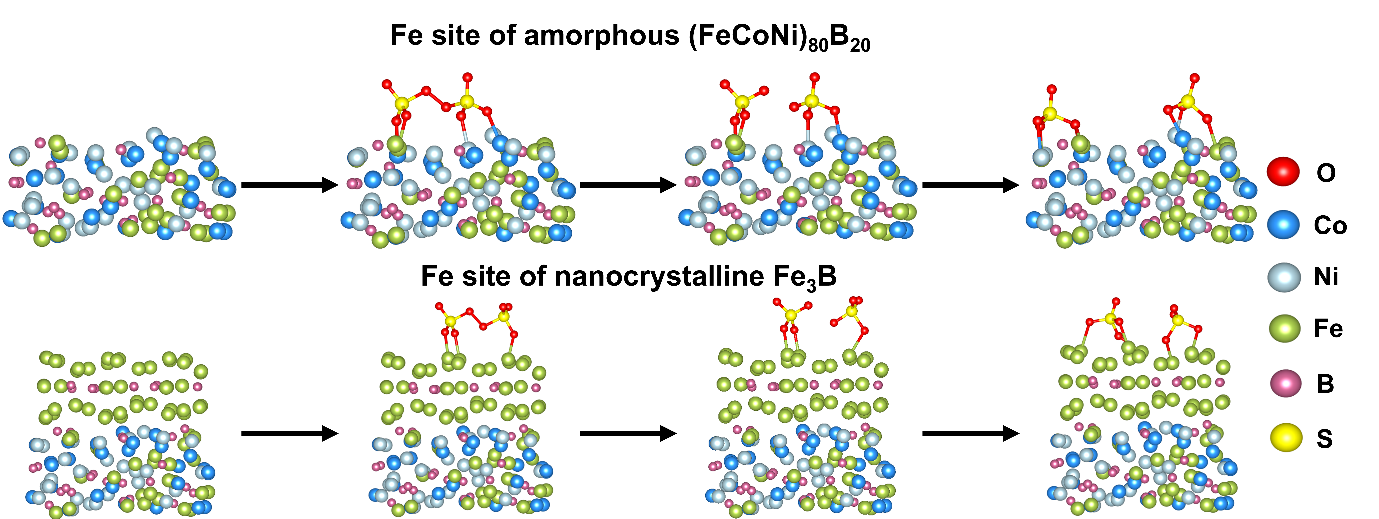


# **Fig. S32** The surface adsorption process of AOPs for Fe site of amorphous (FeCoNi)_80_B_20_ and nanocrystalline Fe_3_B


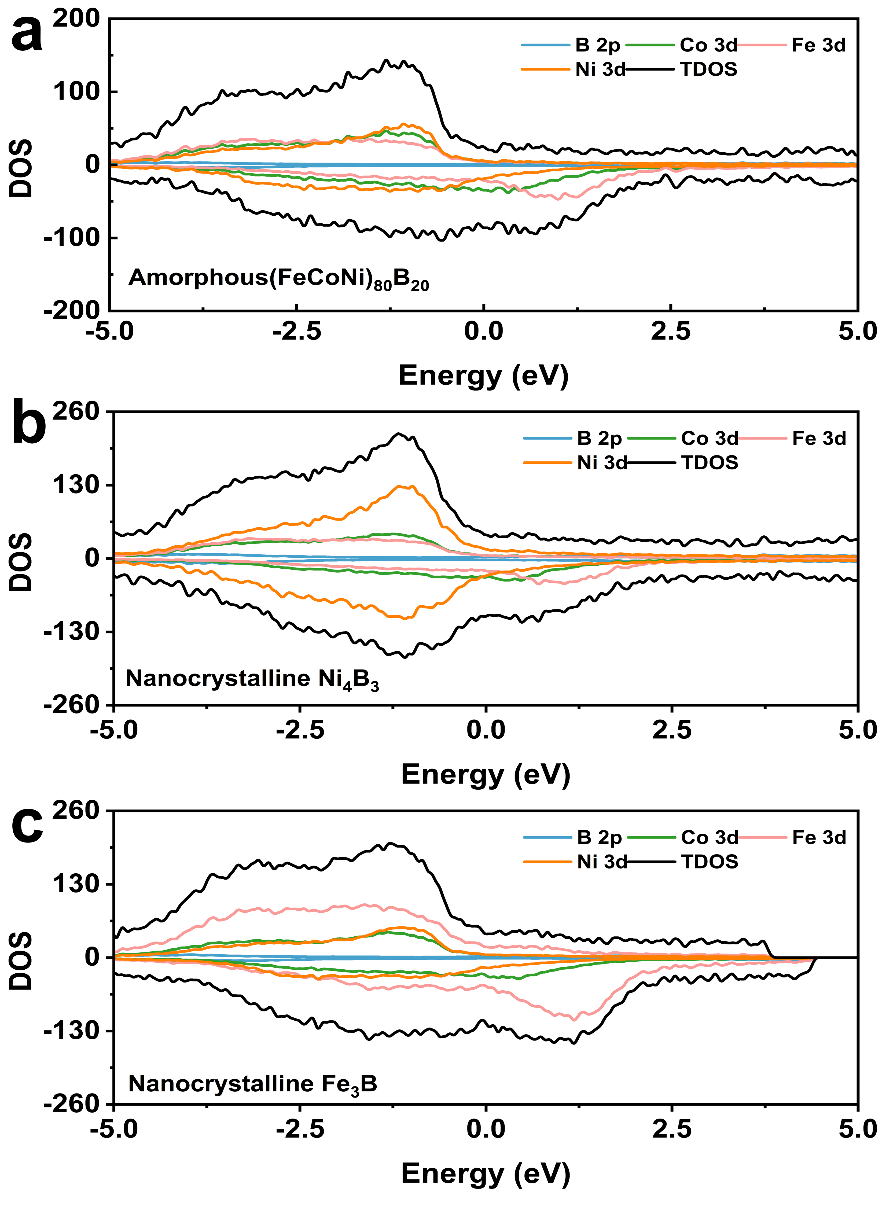


# **Fig. S33** Partial density of states (pDOS) for amorphous **a** (FeCoNi)_80_B_20_, **b** nanocrystalline Ni_4_B_3_ and **c** Fe_3_B. The pDOS analysis further indicated that Fe, Co, and Ni sites collectively regulated the adsorption characteristics of (FeCoNi)_80_B_20_, thereby facilitating intermediate desorption in both catalytic reactions


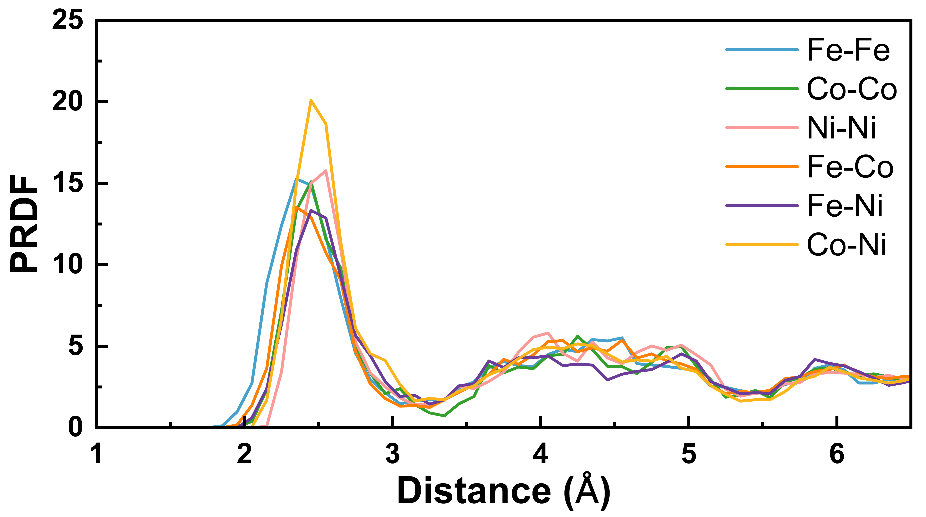


# **Fig. S34** Partial radial distribution function (PRDF) between Fe, Co and Ni of the (FeCoNi)_80_B_20_. The analysis of the first PRDF peak reveals the specific coordination number for each sample

# **Table S1** Comparison of *k_obs_* and reusability times for various catalysts

| **Materials** | **Reusability (times)** | ***k_obs_* (min^-1^)** | **References** |
| --- | --- | --- | --- |
| Fe_3_O_4_ | 6 | 0.019 | [S1] |
| FeSiB | 3 | 0.16 | [S2] |
| FeSiB | 4 | 0.519 | [S3] |
| FeSiB | 8 | 0.8 | [S4] |
| FeSiB | 15 | 1.34 | [S5] |
| FeSiB | 20 | 0.64 | [S6] |
| FeSiB | 23 | 0.25 | [S7] |
| FeSiB/Cu | 8 | 0.217 | [S8] |
| FeBY | 11 | 0.011 | [S4] |
| FeMoB | 15 | 0.36 | [S9] |
| CoMoB | 20 | 2.31 | [S10] |
| FeCuBP | 4 | 0.4 | [S2] |
| FeSiBNb | 10 | 0.183 | [S11] |
| FeCoNiB | 30 | 1.366 | This work |
| FeAlCoNiB | 5 | 0.55 | [S12] |
| FeCoCrMoCBY | 50 | 0.57 | [S13] |

# **Table S2** Comparison of OER overpotential and Tafel slope for various catalysts

| **Materials** | **Overpotential at**  **10 mA cm^-2^ (mV)** | **Tafel slope**  **(mV dec^-1^)** | **References** |
| --- | --- | --- | --- |
| IrO_2_ | 260 | 45.0 | [S14] |
| RuO_2_ | 275 | 110.7 | [S15] |
| NiFe LDH | 300 | 40.0 | [S14] |
| FeCo LDH | 331 | 85.0 | [S16] |
| FeNiB | 319 | 56.0 | [S17] |
| FeCoB | 315 | 26.0 | [S18] |
| FeNiMoB | 227 | 35.0 | [S19] |
| CoMoO_4_ | 312 | 56.0 | [S20] |
| FeCoNiMoPB | 281 | 36.49 | [S21] |
| FeNi | 335 | 53.5 | This work |
| FeCoNi | 288 | 45.3 | This work |
| FeCoNiB | 276 | 53.45 | This work |

**Table S3** The ICP-OES results of the electrolyte after 250 h water splitting stability test at 200 mA cm^-2^

| **Element** | **Concentration (µg/mL)** |
| --- | --- |
| Fe | 21.39 |
| Co | 29.70 |
| Ni | 26.63 |

**Table S4** The ICP-OES results of the electrolyte after AOPs and synchronized process

| **Reactions** | **Elements** | **Precipitation rate (µg/mL/min）** |
| --- | --- | --- |
| AOPs | Fe | 0.239 |
| AOPs | Co | 0.335 |
| AOPs | Ni | 0.346 |
| synchronized process | Fe | 1.015 |
| synchronized process | Co | 1.492 |
| synchronized process | Ni | 1.5265 |

**Table S5** Comparison of water splitting performance at high current density of medium/high entropy amorphous alloys prepared by various methods

| Material | Current density  (mA cm^-2^) | Voltage  (V) | Prepared methods | References |
| --- | --- | --- | --- | --- |
| NiCoFeP @NiCoFe-LDH | 1000 | 1.86 | Hydrothermal synthesis+Annealing | [S22] |
| AC-NP-CuNiCo | 10 | 1.53 | Melt-spinning method+Dealloying | [S23] |
| RuVCoCuZnW | 500 | 1.66 | Flash joule heating device | [S24] |
| AC-HEA-CuAgAuIrRu | 10 | 1.49 | Melt-spinning method+Dealloying | [S25] |
| FeCoNiCuMoB | 10 | 1.48 | Magnetron sputtering | [S26] |
| Fe-NiWB | 500 | 1.55 | Electroless plating | [S27] |
| NiNbIrPt | 10 | 1.51 | Melt-spinning method+Dealloying | [S28] |

# **Supplementary References**

[S1] P. V. Nidheesh, R. Gandhimathi, S. Velmathi, N. S. Sanjini. Magnetite as a heterogeneous electro fenton catalyst for the removal of rhodamine b from aqueous solution. RSC Adv. **4**(11), 5698-5708 (2014). https://doi.org/10.1039/C3RA46969G

[S2] P. Zhang, Y. a. Zhu, Y. Zhang, T. Lu, Y. Pan. Heterogeneously structured fecubp amorphous–nanocrystalline alloy with excellent dye degradation efficiency. Appl. Phys. A. **127**(5), 330 (2021). https://doi.org/10.1007/s00339-021-04485-x

[S3] Z. Jia, X. Duan, P. Qin, W. Zhang, W. Wang et al., Disordered atomic packing structure of metallic glass: Toward ultrafast hydroxyl radicals production rate and strong electron transfer ability in catalytic performance. Adv. Funct. Mater. **27**(38), 1702258 (2017). https://doi.org//10.1002/adfm.201702258

[S4] P. Liu, J. L. Zhang, M. Q. Zha, C. H. Shek. Synthesis of an Fe rich amorphous structure with a catalytic effect to rapidly decolorize azo dye at room temperature. ACS Appl. Mater. Interfaces. **6**(8), 5500-5505 (2014). https://doi.org/10.1021/am501014s

[S5] Y.-X. Ge, P.-Y. Zhu, Y. Yu, L.-C. Zhang, C. Zhang et al., Remarkably enhanced fenton-like catalytic activity and recyclability of fe-based metallic glass by alternating magnetic field: Mechanisms and industrial applications. J. Mater. Chem. A. **10**(43), 23314-23322 (2022). https://doi.org/10.1039/D2TA06216J

[S6] Z. Jia, X. Duan, W. Zhang, W. Wang, H. Sun et al., Ultra-sustainable Fe_78_Si_9_B_13_ metallic glass as a catalyst for activation of persulfate on methylene blue degradation under uv-vis light. Sci. Rep. **6**(1), 38520 (2016). https://doi.org/10.1038/srep38520

[S7] Z. Jia, J.-L. Jiang, L. Sun, L.-C. Zhang, Q. Wang et al., Role of boron in enhancing electron delocalization to improve catalytic activity of fe-based metallic glasses for persulfate-based advanced oxidation. ACS Appl. Mater. Interfaces. **12**(40), 44789-44797 (2020). https://doi.org/10.1021/acsami.0c13324

[S8] J. Wei, Z. Zheng, L. Huang, Z. Qiu, Q. Xia et al., Effective removal of orange ii dye by porous fe-base amorphous/cu bimetallic composite. Colloids Surf. A Physicochem. Eng. Asp. **656**, 130388 (2023). https://doi.org//10.1016/j.colsurfa.2022.130388

[S9] Y.-n. Chen, S. Xiao, Y. Yang, C. Su, C. Dai et al., The rapid degradation of dye wastewater utilizing mofeb amorphous alloy wires. Colloids Surf. A Physicochem. Eng. Asp. **726**, 137831 (2025). https://doi.org//10.1016/j.colsurfa.2025.137831

[S10] M. Tang, L. Lai, D. Ding, T. Liu, W. Kang et al., Rapid degradation of direct blue dye by co-based amorphous alloy wire. J. Non-Cryst. Solids. **576**, 121282 (2022). https://doi.org//10.1016/j.jnoncrysol.2021.121282

[S11] J. C. Wang, Z. Jia, S. X. Liang, P. Qin, W. C. Zhang et al., Fe_73.5_Si_13.5_B_9_Cu_1_Nb_3_ metallic glass: Rapid activation of peroxymonosulfate towards ultrafast eosin y degradation. Mater. Des. **140**, 73-84 (2018). https://doi.org//10.1016/j.matdes.2017.11.049

[S12] S. Xie, X. Liao, X. Zeng, H. Yang, L. He. An iron-based high-entropy alloy with highly efficient degradation for p-nitrophenol. Acta Metall. Sin. (Engl. Lett.). **35**(10), 1653-1664 (2022). https://doi.org/10.1007/s40195-022-01402-w

[S13] W. Luo, Q. Chen, L. Ji, X. Peng, G. Huang. Synergistic effect of various elements in Fe_41_Co_7_Cr_15_Mo_14_C_15_B_6_Y_2_ amorphous alloy hollow ball on catalytic degradation of methylene blue. J. Rare Earths. **40**(4), 605-615 (2022). https://doi.org//10.1016/j.jre.2021.02.003

[S14] D. Friebel, M. W. Louie, M. Bajdich, K. E. Sanwald, Y. Cai et al., Identification of highly active fe sites in (ni,fe)ooh for electrocatalytic water splitting. J. Am. Chem. Soc. **137**(3), 1305-1313 (2015). https://doi.org/10.1021/ja511559d

[S15] Z. Kou, Y. Yu, X. Liu, X. Gao, L. Zheng et al., Potential-dependent phase transition and mo-enriched surface reconstruction of γ-coooh in a heterostructured co-mo2c precatalyst enable water oxidation. ACS Catal. **10**(7), 4411-4419 (2020). https://doi.org/10.1021/acscatal.0c00340

[S16] B. Zhang, X. Zheng, O. Voznyy, R. Comin, M. Bajdich et al., Homogeneously dispersed multimetal oxygen-evolving catalysts. Science. **352**(6283), 333-337 (2016). https://doi.org/doi:10.1126/science.aaf1525

[S17] H.-l. Li, Y.-y. Wang, C.-m. Liu, S.-m. Zhang, H.-f. Zhang et al., Enhanced oer performance of nifeb amorphous alloys by surface self-reconstruction. Int. J. Hydrogen Energy. **47**(48), 20718-20728 (2022). https://doi.org//10.1016/j.ijhydene.2022.04.200

[S18] L. Xiao, J. Gao, X. Yao, X. Dong, F. Yang et al., Unraveling the role of nanocrystalline in amorphous nanoporous fecob catalysts for turning oxygen evolution activity and stability. Appl. Surf. Sci. **640**, 158395 (2023). https://doi.org//10.1016/j.apsusc.2023.158395

[S19] B. Li, S.-D. Jiang, Q. Fu, R. Wang, W.-Z. Xu et al., Tailoring nanocrystalline/amorphous interfaces to enhance oxygen evolution reaction performance for feni-based alloy fibers. Adv. Funct. Mater. **35**(2), 2413088 (2025). https://doi.org//10.1002/adfm.202413088

[S20] M. Q. Yu, L. X. Jiang, H. G. Yang. Ultrathin nanosheets constructed comoo4 porous flowers with high activity for electrocatalytic oxygen evolution. Chem. Commun. **51**(76), 14361-14364 (2015). https://doi.org/10.1039/C5CC05511C

[S21] H. Chen, Y. Xin, Y. Wu, J. Jiang, D. Ding et al., Boosting the activity and stability of self-supporting feconimopb amorphous alloy for oxygen evolution. J. Alloys Compd. **947**(169478 (2023). https://doi.org//10.1016/j.jallcom.2023.169478

[S22] X. Fu, H. Liao, Z. Zhang, Y. Zheng, J. Lu et al., Medium-entropy heterostructure of crystalline nicofep @ amorphous nicofe-ldh for industrial-current density and ultrastable overall water splitting. Chem. Eng. J. **505**(159520 (2025). https://doi.org//10.1016/j.cej.2025.159520

[S23] M. Liu, S. Ning, D. Xiao, Y. Zhang, J. Han et al., Amorphous/crystalline heterostructured nanoporous high-entropy metallic glasses for efficient water splitting. Mater. Futures. **4**(2), 025303 (2025). https://doi.org/10.1088/2752-5724/add415

[S24] K. Li, W. Qiao, N. Li, M. Lü, C. Gu et al., Ultrafast microwave construction of stabilized ruco alloys for overall water splitting. J. Chem. Phys. **162**(24), (2025). https://doi.org/10.1063/5.0268895

[S25] X. Yu, X. Gong, H. Qiao, X. Liu, C. Ma et al., Amorphous-crystalline heterostructured nanoporous high-entropy alloys for high-efficiency ph-universal water splitting. Small Methods. **8**(10), 2400793 (2024). https://doi.org//10.1002/smtd.202400793

[S26] S. Wang, H. Yan, W. Huo, A. Davydok, M. Zając et al., Engineering multiple nano-twinned high entropy alloy electrocatalysts toward efficient water electrolysis. Appl. Catal. B: Environ. **363**(124791 (2025). https://doi.org//10.1016/j.apcatb.2024.124791

[S27] Y. Liao, L. Li, J. Zhang, Y. Chen, S. Luo et al., Colloid-mediated synthesis of hierarchically porous amorphous catalyst for durable industrial-scale water electrolysis. Adv. Mater. e16751 (2025). https://doi.org//10.1002/adma.202516751

[S28] Y. Zhang, R. Li, X. Wang, Q. Guo, Q. Zhang et al., Surface active-site engineering of low-noble-metal-alloyed metallic glass catalyst for boosting water electrolysis. Adv. Funct. Mater. **34**(51), 2410379 (2024). https://doi.org//10.1002/adfm.202410379
